# Investigation of Enzymes in the Phthalide Biosynthetic Pathway in *Angelica sinensis* Using Integrative Metabolite Profiles and Transcriptome Analysis

**DOI:** 10.3389/fpls.2022.928760

**Published:** 2022-07-01

**Authors:** Wei-Meng Feng, Pei Liu, Hui Yan, Guang Yu, Sen Zhang, Shu Jiang, Er-Xin Shang, Da-Wei Qian, Jin-Ao Duan

**Affiliations:** Jiangsu Key Laboratory for High Technology Research of TCM Formulae, Jiangsu Collaborative Innovation Center of Chinese Medicinal Resources Industrialization, National and Local Collaborative Engineering Center of Chinese Medicinal Resources Industrialization and Formulae Innovative Medicine, Nanjing University of Chinese Medicine, Nanjing, China

**Keywords:** *Angelica sinensis*, phthalides biosynthetic pathway, transcriptome, regulation mechanism, prokaryotic expression

## Abstract

The roots of *Angelica sinensis* (Oliv.) Diels are well known for their efficacy in promoting blood circulation. Although many studies have indicated that phthalides are the main chemical components responsible for the pharmacological properties of *A. sinensis,* the phthalide biosynthetic pathway and enzymes that transform different phthalides are still poorly understood. We identified 108 potential candidate isoforms for phthalide accumulation using transcriptome and metabolite profile analyses. Then, six enzymes, including phospho-2-dehydro-3-deoxyheptonate aldolase 2, shikimate dehydrogenase, primary amine oxidase, polyphenol oxidase, tyrosine decarboxylase, and shikimate O-hydroxycinnamoyl transferase, were identified and proven to be involved in phthalide accumulation by heterologously expressing these proteins in *Escherichia coli*. We proposed a possible mechanism underlying phthalide transformation and biosynthetic pathways in *A. sinensis* based on our findings. The results of our study can provide valuable information for understanding the mechanisms underlying phthalide accumulation and transformation and enable further development of quality control during the cultivation of *A. sinensis*.

## Introduction

The *Angelica sinensis* (Oliv.) Diels is a high-altitude plant found in the marginal region of the Qinghai-Tibet Plateau. The roots of *A. sinensis* have a long history of being widely used in Traditional Chinese Medicine for treating various gynecological conditions (Fang et al., 2012). As one of the most frequently used Chinese medicinal materials in clinical practice, the germplasm, cultivation, harvesting, medicinal components, and pharmacological activities of *A. sinensis* have received extensive attention and continuous research. The effectiveness and therapeutic mechanisms of the *A. sinensis* are being explored and investigated. Over 180 phytochemicals have been identified in *A. sinensis* grown under high-altitude conditions, including phthalides, phenylpropanoids, terpenoids, alkynes, and alkaloids (Zou et al., 2018). Among these, phthalides were selected as marker compounds for quality control and pharmacokinetic studies of *A. sinensis* (Wei and Huang, 2015). The extensive cultivation of the plant in different areas across the country revealed the problem of early flowering in *A. sinensis*, which causes root burn and reduction of plant oils, seriously affecting its quality and yield. Previous studies (Li et al., 2020c) mainly focused on the influencing factors and compound content analysis of the early flowering *A. sinensis*. However, the molecular mechanism underlying the change in phthalides during the early flowering of *A. sinensis* is still unclear. Therefore, identifying the key enzymes that affect the synthesis and accumulation of phthalides in the early flowering process provides important guidance in cultivating *A. sinensis*.

Phthalides are among the most important active ingredients in volatile plant oils and a characteristic component of important natural compounds from Umbelliferae plants, such as *A. sinensis* and *Ligusticum chuanxiong* (Tang et al., 2021). Recent research has indicated that phthalides are the main chemical components related to the bioactivity and pharmacological properties of *A. sinensis,* such as anti-asthma, anti-convulsant, inhibition of platelet aggregation, and enhancement of blood flow (Yi et al., 2009). In particular, n-Butylphthalide was approved by the State Food and Drug Administration of China in 2005 as a modern drug for treating ischemic strokes (Zou et al., 2018). Moreover, butylphthalide and ligustilide that show insecticidal activity against the B- and Q-biotype females of *Bemisia tabaci* (Chae et al., 2011) and *Drosophila melanogaster* (Miyazawa et al., 2004) are potential alternatives to conventional arthropod control products. This has received considerable attention from the public because they are relatively safe and poses fewer risks to the environment (Isman, 2006).

Phthalides have been recognized for their broad-spectrum biological activities. As important index components in *A. sinensis*, it is important to determine the factors that regulate phthalide accumulation (Karmakar et al., 2014). First, the expression of enzymes in the phthalide biosynthetic pathway is considered one of the key factors. Elucidating the biosynthesis of phthalides began with the structural determination of mycophenolic acid, a phthalide fragment derived from the polyketide pathway (Birch et al., 1958). Thereafter, researchers identified the biogenetic origin of butylphthalide by conducting feeding experiments to explain the formation of ligustilide in *Levisticum officinale* and determined that the alkylphthalide has polyketide precursors (Mitsuhashi and Nomura, 1966). Although it has been explored, phthalide biosynthesis, especially the interconversion mechanism between different phthalides, remains unclear and needs further investigation.

The compound content of medicinal plants varies at different developmental stages. The normal growth cycle of *A. sinensis* is 3 years, with seedlings raised in the first year, drug-forming in second year, and bolting and flowering in the third year. However, 20–30% of plants bolt and flower in the second year (Yu et al., 2019). The early bolting and flowering have a significant effect on the accumulation of secondary metabolites of *A. sinensis*, especially the reduction of volatile oil components mainly composed of phthalides. The early bolting and flowering significantly reduce the yield and quality of the roots, which seriously affects the medicinal material available on the market and the economic benefits by farmers of medical crops (Li et al., 2021). There are various reasons for early flowering, such as seedling size, environmental temperature, hormones, and microorganisms, all of which may cause early bolting in *A. sinensis*, and hence, the problem of early flowering cannot be solved by fixing a single factor (Li et al., 2020c). Thus, it may be a feasible strategy to increase the phthalide content during the flowering of *A. sinensis* to reduce waste, improve the market supply of medicinal raw materials, and alleviate the economic losses of pharmaceutical farmers. Although early flowering plants cannot be directly introduced into the market as medicinal materials, plants with high medicinal ingredient contents may become new sources of raw active ingredients.

In this study, the ultra-high performance liquid chromatography–tandem mass spectrometry (UHPLC–MS/MS) method was utilized to identify six phthalides, including ligustilide, butylphthalide, butylidenephalide, senkyunolide H, senkyunolide I and senkyunolide A, in the roots of normal flowering and early flowering *A. sinensis* plants. The absolute levels of the six phthalides and the changes in their proportions were analyzed. Illumina MiSeq high-throughput sequencing technology was used to investigate the root transcriptome. We aimed to explore candidate enzymes that positively correlated with phthalide accumulation, followed by an analysis of the content of the six phthalides and their transcriptional expression. The function of the candidate isoforms and the potential phthalide biosynthetic pathways were further determined using quantitative real-time polymerase chain reaction (qRT-PCR) and prokaryotic expression. The results from our study expand the understanding of the changes in the phthalide content between early flowering and normal flowering in *A. sinensis*. This provides insights for developing a new plant variety with a high phthalide level or a characteristic phthalide content.

## Materials and Methods

### Plant Materials, Chemicals, and Reagents

In this study, root samples from the normal flowering (ZC-1 to ZC-6) and early flowering (ZT-1 to ZT-6) fresh *A. sinensis* plants of the plant strain “Mingui No. 1,” were selected as the experimental materials. Samples were collected on August 22, 2018, from Tanchang, Gansu Province (104.14780 E, 34.12113 N, Height- 2,260 m). Dr. Hui Yan from the Nanjing University of Chinese Medicine authenticated the roots. Fresh *A. sinensis* roots were collected, flash-frozen in liquid nitrogen, and transported in dry ice. The samples were stored at −80°C at the Jiangsu Collaborative Innovation Center of Chinese Medicinal Resources Industrialization. The high-throughput sequencing analysis was performed at Entrusted Frasergen Bioinformatics Co., Ltd. (Wuhan, China).

Tissue culture seedlings of *A. sinensis* “Mingui No. 1” were grown in a culture room at 23 ± 1°C under a 14-h photoperiod and 2000 lx. Methyl jasmonate (MeJA; 100 μM; Ho et al., 2020; Lv et al., 2021; Zhou et al., 2021) was added to each flask prior to the solidification of the medium. When cultivation under static conditions for 30 to 40 days and the cotyledons were flattened and the true leaf sprouted, tissue culture seedlings were transferred to MeJA-containing medium for further culture. To verify the candidate isoforms, tissue culture seedlings of *A. sinensis* were collected at the following time points after treatment with MeJA: 0, 24, 48, 72, and 96 h.

Reference Standards, Including Ligustilide, Butylphthalide, Butylidenephalide, Senkyunolide H, Senkyunolide I, and Senkyunolide A (Supplementary Figure S1), all 98% Purified, Were Obtained From Liangwei Biochemical Reagent Ltd. (Nanjing, China).

### Transcriptome Sequencing, Assembly, and Analysis

Two randomly selected plant roots were combined for transcriptome analysis, and each group (ZT and ZC groups) contained three samples for analysis. Libraries were constructed from root mRNA and sequenced using the PacBio Sequel and Illumina HiSeq X Ten PE150 platforms (Illumina, San Diego, CA, United States) by the Frasergen Biotechnology Company (Wuhan, China). Sequencing libraries were generated using the NEB Next^®^ Ultra TM RNA Library Prep Kit (NEB, Ipswich, MA, United States) based on Illumina^®^ manufacturer’s protocols, and index codes were added to attribute sequences to each sample.

Due to the limited genomic information of *A. sinensis*, the full-length transcript sets from the roots of normal and early flowering *A. sinensis* plants by PacBio SMRT three-generation high-throughput sequencing technology were used as the reference isoforms for both subsequent bioinformatics analysis and comparative transcriptomics analysis. Sequencing reads were aligned to the reference isoforms using Tophat2 (v2.1.1) and Bowtie2 (v2.2.2) with default parameters (Li et al., 2020b). The expression of genes and isoforms was quantified using the RSEM software package (RNASeq by Expectation–Maximization v1.3.0). Gene expression differentiation was screened using the following criteria: fold change ≥2 and false discovery rate < 0.05. The differentially expressed genes were subjected to enrichment analysis of Gene Ontology (GO) functions and Kyoto Encyclopedia of Genes and Genomes (KEGG) pathways, with *p* ≤ 0.01 and false discovery rate ≤ 0.05 as the thresholds for both analyses by KOBAS (v3.0; Li et al., 2020a). All transcripts were annotated against the Non-Redundant (NR), GO, and KEGG databases using Diamond (v0.8.33). qRT-PCR analysis was performed to validate the transcriptome data. Correlation analysis was performed by selecting transcripts that were differentially expressed between ZT and ZC groups and were consistent with the trend of phthalide content. The datasets generated during the current study were deposited and are available at the National Center for Biotechnology Information Sequence Read Archive under accession number PRJNA749925 (PRJNA749925).^1^

### Quantification of Phthalides in Root Samples and Meja-Treated Samples

Standard and sample solutions were prepared using an established method, as described previously (Feng et al., 2021). Chromatographic analyses were performed using a Waters Acquity UPLC system (Waters Corp., MA, United States), whereas mass spectromet was conducted using an AB SCIEX Triple Quad 6,500 plus (AB SCIEX Corp., Framingham, MA, United States) with electrospray ionization. The dwell time was automatically set using the MultiQuant software. Raw data were processed using MultiQuant v3.0.2 (AB SCIEX Corp.). A detailed description of the standard solution, chromatographic conditions, and methodology validation were presented in our previous study (Feng et al., 2021).

### RNA Extraction and qRT-PCR

For qRT-PCR analysis, the total RNA of the tissue culture seedlings of *A. sinensis* was extracted at each time point (0, 24, 48, 72, and 96 h) after MeJA treatment. The RNA prep pure plant kit (polysaccharides polyphenolics-rich; Tiangen, Beijing, China) was used for RNA extraction with on-column DNA digestion according to the manufacturer’s protocol. Total RNA (1.5 μg) was reverse transcribed using random primers and conditions described in the EasyScript All in-one First-Strand cDNA Synthesis SuperMix for qPCR (One-Step gDNA Removal; Trans gene, Beijing, China). RNA and cDNA concentrations and purities were estimated using a DS-11 spectrophotometer (DeNovix, Wilmington, DE, United States). qRT-PCR was performed on an ABI 7500 real-time PCR system (Applied Biosystems, Waltham, MA, United States; Plasencia et al., 2016). Relative gene expression was estimated using the housekeeping gene 18S rRNA as a reference according to the 2^−∆∆Ct^ method (Livak and Schmittgen, 2001). Following the cycling stage, product melting curves were generated to ensure the specificity of product formation. All procedures were performed according to the manufacturer’s instructions. Primers used for qPCR are listed in Supplementary Table S1. Two technical replicates were used for each sample, and three samples were analyzed for each group.

### Prokaryotic Expression Function Verification of Key Candidate Isoforms

The full-length cDNA of the seven candidate isoforms was cloned into the pET-28a (+) vector. The recombinant plasmids were transformed into *Escherichia coli* BL21 (DE3) cells to express recombinant proteins. The positive clones were incubated in Luria-Bertani medium in the presence of kanamycin. *E. coli* BL21 (DE3) were grown in 250 ml Erlenmeyer baffle flasks containing 100 ml of Luria-Bertani medium in a rotary shaker at 160 rpm and 37°C. When the optical density of the cultures at 600 nm reached 0.5–0.6, recombinant proteins were expressed in *E. coli* cells following induction by the addition of 0.5 mM isopropyl-β-D-thiogalactoside and incubation overnight at 16°C. After centrifugation at 32000 × *g* for 10 min, the cells were resuspended in 1 ml phosphate buffer saline solution (pH 7.2–7.4). The cells were lysed using an ultrasonic disrupter at 15% power for 3 min, centrifuged at 12000 × *g* for 10 min, and then purified using Ni-NTA affinity chromatography under nature conditions, following the manufacturer’s instructions (Cytiva, Seattle, WA, United States). After purification with Ni-NTA chromatography, the protein sample was analyzed by sodium dodecyl sulfate-polyacrylamide gel electrophoresis (Zhao et al., 2020). The protein thus obtained was used for the subsequent enzymatic reaction.

Centrifugal tubes (1.5 ml) containing 250 μl reaction mixtures, which included 100 μl of crude enzyme solution, 110 μl of 25 mM Tris–HCl (pH 7.5), 10 μl of 100 mM magnesium chloride hexahydrate (6H_2_O MgCl_2_), 10 μl of 50 mM DL-dithiothreitol, and 20 μl of various phthalide (5 mg/ml)-methanol extracts, were incubated for 1 h at 30°C in the dark. Thereafter, the mixtures were centrifuged at 12000 × *g* for 10 min prior to UHPLC–MS/MS analysis (Xiong et al., 2020).

### Statistical Analysis

A Student’s *t*-test for the phthalide content and relative expression analysis was performed using SPSS v21.0. The results, presented as the mean ± standard deviation, were processed and optimized using GraphPad Prism 7.0.

## Results

### Targeted Metabolite Profile Analysis of Phthalide Contents in Roots of Normal and Early Flowering *Angelica sinensis*

UHPLC–MS/MS was used to simultaneously determine six phthalide markers in 12 samples of *A. sinensis* (Supplementary Table S2). The most selective and specific transition was chosen for multiple reaction monitoring (MRM) determination, and all the MRM parameters are provided in Supplementary Table S3. The UHPLC method was validated by assessing the linearity, precision, stability, limit of detection, limit of quantification, and recovery (Feng et al., 2021).

**Figure 1:**
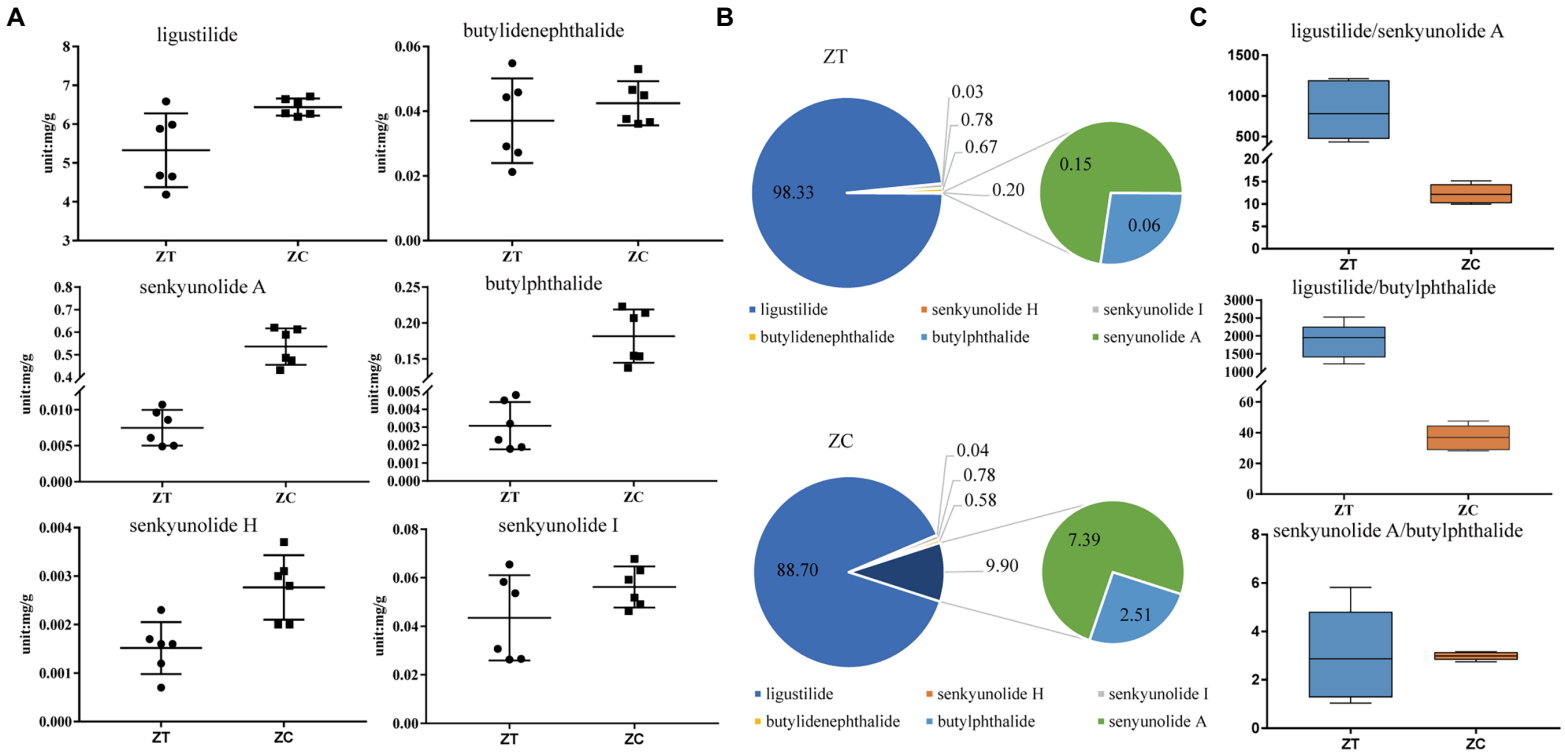
Levels of the tested components across samples. **(A)** Content levels of the six phthalides in ZT and ZC samples; **(B)** The percentage of each phthalide level in the total phthalide of ZT and ZC samples; **(C)** The diagnostic ratios of three characteristic phthalides with large differences, ligustilide/butylphthalide, ligustilide/senkyunolide A, and senkyunolide A/butylphthalide, in ZT and ZC samples.

Principal component analysis was performed to determine variations in the metabolites. Overall, the metabolite profiles of the two groups of samples differed substantially. The first principal component accounted for 68.4% of the metabolic variance between the ZT and ZC groups (Supplementary Figure S2). The level of ligustilide was found to be the highest among the six phthalides in the ZC (6.191–6.713 mg/g) and ZT samples (4.186–6.586 mg/g). The total amount of the six phthalides was 5.421 and 7.258 mg/g in the ZT and ZC samples, respectively. The average levels of ligustilide, butylphthalide, senkyunolide H, and senkyunolide A were found to be significantly higher in the ZC samples (6.439 ± 0.22 mg/g, 181.6 ± 37.08, 2.795 ± 0.66, and 535.5 ± 81.09 μg/g, respectively) compared to those in the ZT samples (5.327 ± 0.95 mg/g, 3.088 ± 1.30, 1.516 ± 0.52, and 7.495 ± 2.49 μg/g, respectively; *p* < 0.05). However, the levels of senkyunolide I and butylidenephalide were not significantly different between the ZC (56.20 ± 8.45 and 42.47 ± 6.82 μg/g, respectively) and ZT samples (43.51 ± 17.60 and 37.07 ± 13.10 μg/g, respectively; Figure 1A; Supplementary Table S2).

Through the absolute quantification of a single component, the levels of all six phthalides were lower in the ZT samples than those in the ZC samples (Figure 1). Moreover, the ratio of each phthalide level to the total phthalide level were distorted in the ZT and ZC samples (Figure 1B). The ratios of senkyunolide H, senkyunolide I, and butylidenephalide showed no obvious changes between the ZC (0.04, 0.78, and 0.58%, respectively) and ZT samples (0.03, 0.78, and 0.67%, respectively). However, the ratio of butylphthalide to senkyunolide A was significantly higher in the ZC samples (2.51 and 7.39%, respectively) than that in the ZT samples (0.06 and 0.15%, respectively; *p* < 0.05). The ratio of ligustilide was significantly higher in the ZT samples (98.33%) than that in the ZC samples (88.70%; *p* < 0.05). We then selected three characteristic phthalides with large differences for the diagnostic ratio analysis (Figure 1C). The diagnostic ratios of ligustilide/butylphthalide, ligustilide/senkyunolide A, and senkyunolide A/butylphthalide were 1884.01, 813.63, and 3.07, respectively, in the ZT samples and 36.94, 12.31, 2.97, respectively, in the ZC samples. These results reflect the change in the composition ratio and the difference in the amounts of the phthalides.

### Differential Transcriptomic Analysis in Roots of Normal and Early Flowering *Angelica sinensis*

**Figure 2:**
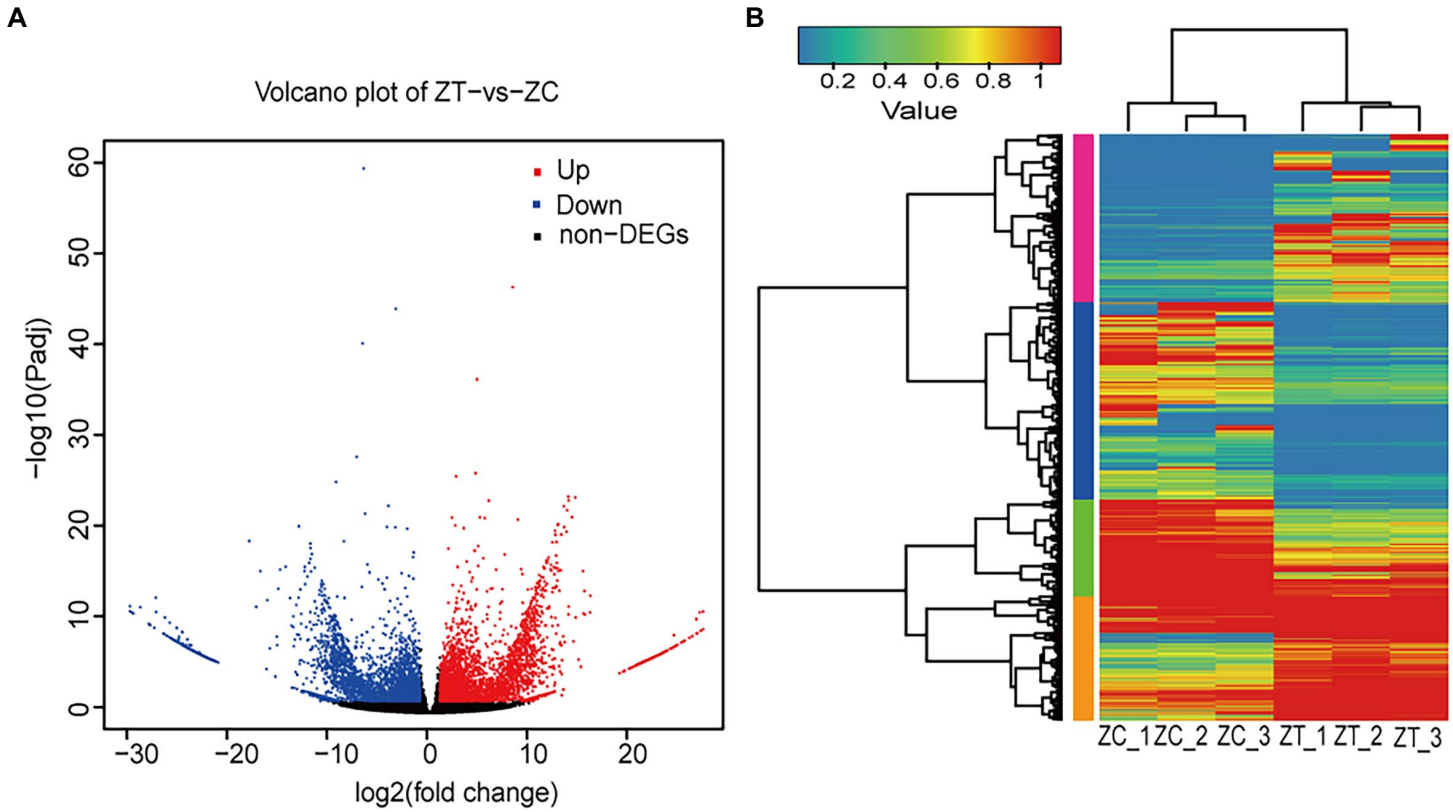
The Volcano plot and Cluster plot of the differential expression isoforms in ZC and ZT samples [**(A)** Volcano plot; **(B)** Cluster plot. ZC: root samples from normal flowering plant; ZT: root samples from early flowering plant].

The RNA-seq yielded 41.36 Gb of clean data, with an average of 6.89 Gb for each sample, with 90.74% of bases scoring > Q30 (Supplementary Table S4). A total of 91,519 isoforms were obtained after assembly. The N50 length obtained was approximately 1,631 bp for the ZC samples and 1904 bp for the ZT samples. The transcriptome data results were validated by qRT-PCR, including five highly expressed genes in ZT (Supplementary Figures S3A,B) and five highly expressed genes in ZC (Supplementary Figures S3C,D). The results showed that the expression levels of the transcriptome were generally consistent with the gene expression trends detected by qRT-PCR, which proved that the transcriptome sequencing results were reliable. We performed functional annotation of the isoforms using various databases, including NR, Swiss-Prot, KEGG, Clusters of Orthologous Genes (COG), EuKaryotic Orthologous Groups (KOG), and GO. Gene expression was estimated using fragments per kilobase of exon per million fragments mapped (FPKM). To identify the differentially expressed genes (isoforms) relevant to phthalide components, we compared the FPKM values of each isoform in ZC to those in ZT samples and retained the isoforms with fold change >2 and a false discovery rate correction set at *p* < 0.05 (Livak and Schmittgen, 2001). The volcano plot was showed that there were 8,824 different isoforms, including 4,455 upregulated and 4,369 downregulated isoforms, found using ZT as the control (Figure 2A). The cluster plot was showed that the expression of isoforms in the two group has a difference in four subclusters (Figure 2B). There was a high expression of ZC sample in the subcluster_1 and subcluster_4, and a low expression of ZC sample in the subcluster_2 and subcluster_3 (Supplementary Figure S4). Then, the GO and KEGG enrichment analysis were performed on the differential isoforms.

A GO enrichment analysis was conducted to identify the biological functions of the upregulated and downregulated isoforms obtained from the different combinations (Supplementary Figure S5). The significantly different isoforms were enriched in terms that are divided into three categories: biological process, cellular component, and molecular function. We observed that the isoforms were enriched in GO terms such as metabolic processes, cellular processes, cells, cell parts, binding, and catalytic activity. The significant enrichment of cell and metabolic processes may be related to the early bolting phenomenon, which is mainly manifested in the rapid growth and development of plants and significant changes in the components of medicinally active ingredients. A KEGG enrichment analysis was also performed (Supplementary Figure S6). Significant enrichment was obtained with functions such as carbon metabolism, starch and sucrose metabolism, biosynthesis of amino acids, plant hormone signal transduction, and the mitogen activated protein kinase signaling pathway. KEGG enrichment analysis also involved primary and secondary metabolism and growth and development hormone regulation.

**Figure 3:**
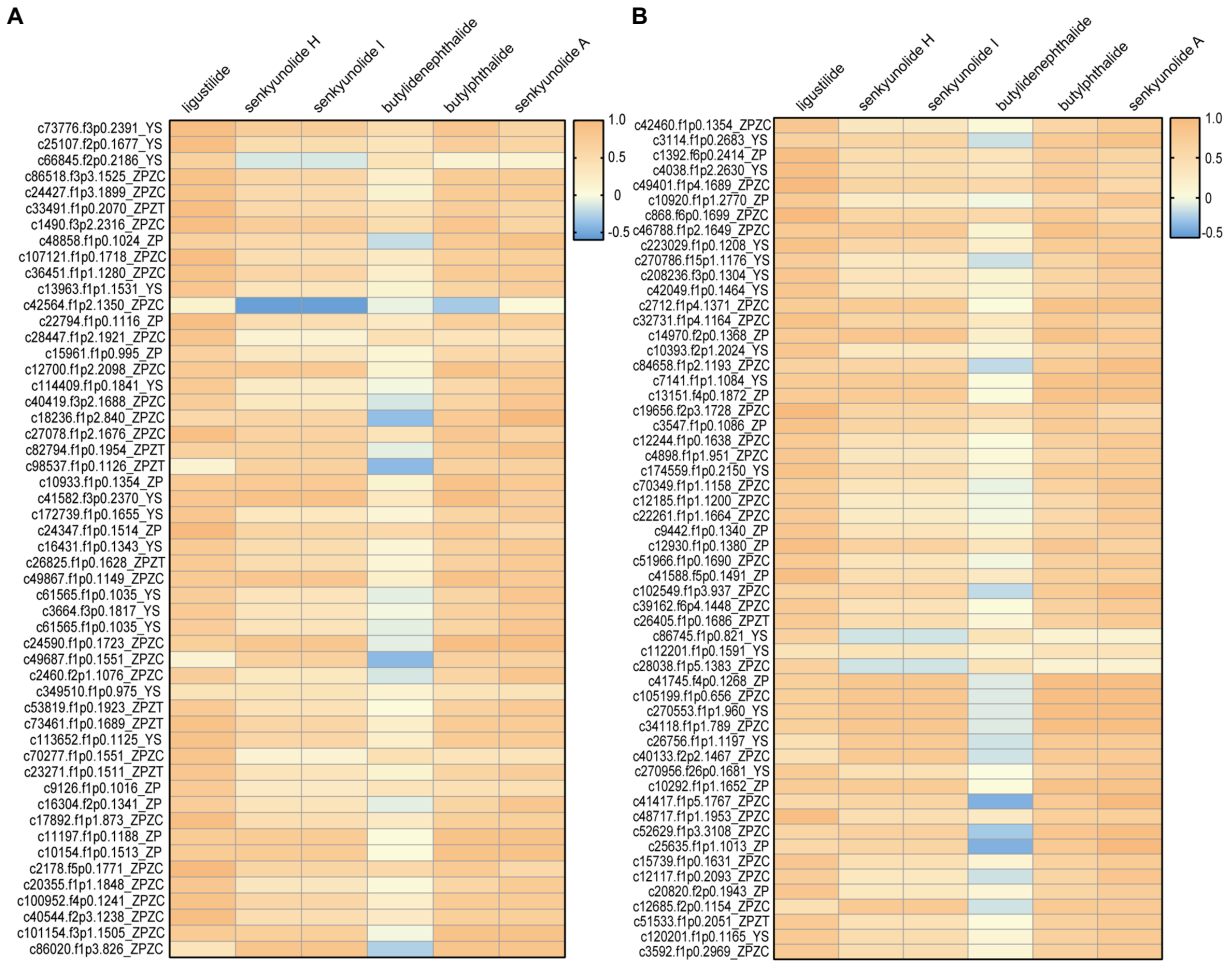
The 108 isoforms that exhibited a significant positive/negative correlation with phthalide accumulation. **(A)** 1-54; **(B)** 55-108.

A relationship analysis was conducted to reveal a Spearman correlation between isoform expression and phthalide content (Supplementary Table S5). Due to the higher content of phthalide in ZC, transcripts with higher expression levels in ZC than ZT were selected for further analysis. Correlation analysis indicated that 57 enzymes had significant positive correlations with phthalide accumulation (Supplementary Table S6). Based on the results of the correlation analysis and isoform function annotation, 108 isoforms were used as the key candidate isoforms for further verification experiments (Figure 3).

### Validation of the Potential Regulatory Role of Key Candidate Isoforms in the Differential Phthalides Content

**Figure 4:**
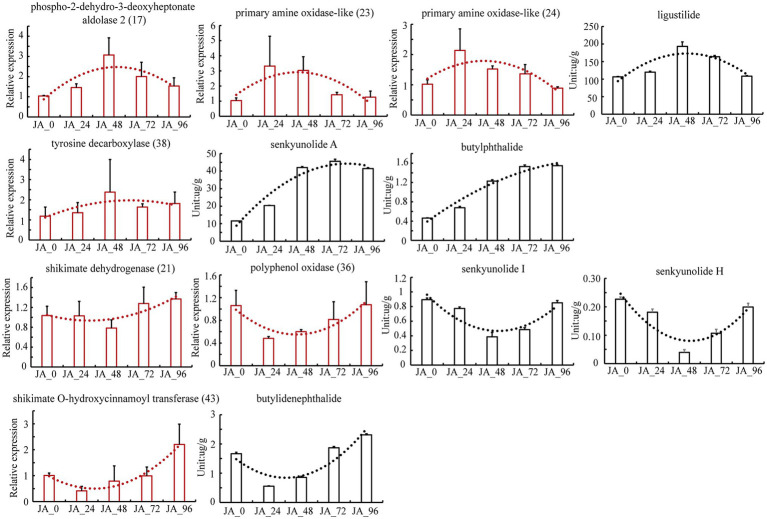
The levels and relative expression of the tested components and isoforms across samples after treatment with methyl jasmonate.

UHPLC–MS/MS was applied to simultaneously determine six markers in tissue culture seedlings of *A. sinensis* after MeJA treatment (Supplementary Table S7). There was an upward trend in the average butylphthalide, and senkyunolide A levels after MeJA treatment. There was an upward trend in the average ligustilide level during 0-48 h MeJA treatment, but a downward trend during 48-96 h MeJA treatment. In contrast, the average levels of butylidenephthalide, senkyunolide I, and senkyunolide H had a downward trend during 0-48 h MeJA treatment, but an upward trend during 48-96 h MeJA treatment. The expression of 108 candidate isoforms was determined using qPCR. This trend in the expression of the seven isoforms was consistent with that of the corresponding phthalide level (Figure 4; Supplementary Table S8). To ensure the reliability of the results, actin was also used as the housekeeping gene in addition to the 18S rRNA gene to verify the selected isoforms again (Supplementary Figure S7). The housekeeping genes 18S rRNA and actin were proved to be stably expressed in the seeds of *A. sinensis* treated with MeJA at different times, based on analysis using BestKeeper software (Pfaffl et al., 2004). The candidate isoforms of phospho-2-dehydro-3-deoxyheptonate aldolase 2 (17) and primary amine oxidase-like (23, 24) may lead to changes in the ligustilide levels, whereas tyrosine decarboxylase (38) may be the reason for the changes in the senkyunolide A levels. The candidate isoforms of shikimate dehydrogenase (21) and polyphenol oxidase (36) are associated with changes in senkyunolide I, senkyunolide H, and shikimate O-hydroxycinnamoyl transferase (43), which showed a trend toward changes in the levels of butylidenephthalide.

### Functional Characterization of Key Candidate Isoforms

**Figure 5:**
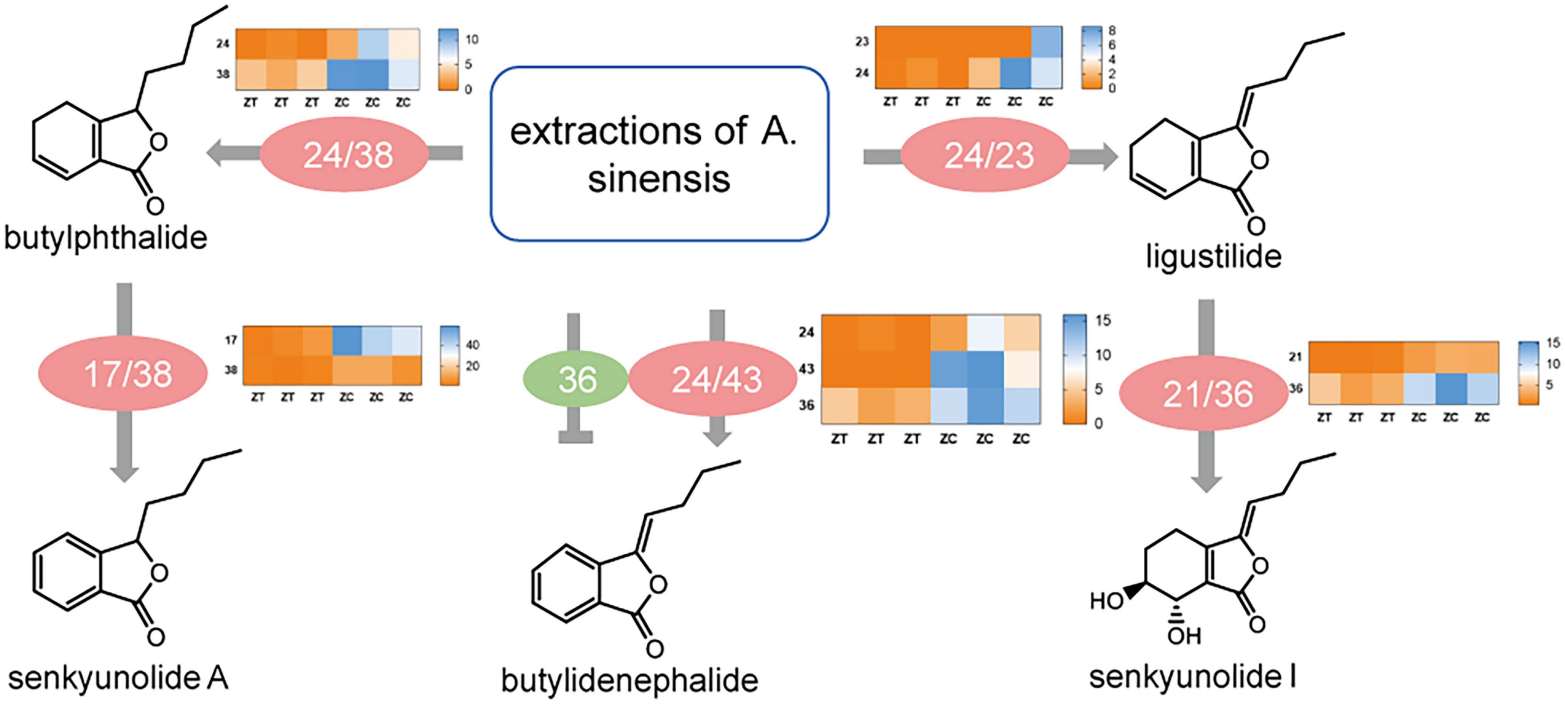
Schematic model of the function of key candidate isoforms in phthalide accumulation and transformation and heatmap of the differential expression of genes involved in phthalide accumulation in ZT and ZC samples. ZC, root samples from normal flowering plant; ZT, root samples from early flowering plant.

To examine these enzymes *in vitro*, we expressed the seven isoforms in *E. coli,* and the proteins were extracted and purified (Supplementary Figure S8). The extracts of *A. sinensis* were incubated with enzymes and subjected to UHPLC–MS/MS analysis. In accordance with *in vitro* experimental results (Supplementary Figure S9; Figure 5), 17 increased butylphthalide levels, whereas 21 increased enkyunolide H levels but decreased butylidenephthalide levels. In addition, 21 and 23 can increase ligustilide levels, 24 can increase the levels of ligustilide, butylidenephthalide, and senkyunolide A, 36 can increase senkyunolide I levels, 38 can increase the levels of senkyunolide A and butylphthalide, and 43 can increase butylidenephthalide levels. However, whether these enzymes are indispensable in the phthalide biosynthetic pathway is not certain and would require knockout experiments to verify this. The data presented here show that overexpression of these seven enzymes had an important influence on the accumulation of phthalides.

## Discussion

*Angelica sinensis* has a long history of use as a traditional herbal medicine and spice in food processing. Hence, analyzing the characteristic components of *A. sinensis* can contribute to its better application in real life. As the plant develops and grows, the effective components continue to be synthesized and are accumulated, and the ratio of the different components is maintained in a balance. However, early flowering results in a decrease in the content of these components, and the ratio becomes distorted. The analysis of the metabolite components of the ZT and ZC samples reveals that the levels of ligustilide, senkyunolide H, senkyunolide I, butylidenephthalide, butylphthalide, and senkyunolide A in the ZT samples were reduced compared with those of the ZC samples. Moreover, we should not only pay attention to the absolute amount of the individual biologically active component but also monitor the changes in the proportions of the different components to ensure better quality and yield of medicinal materials and thus better clinical treatment. The diagnostic ratio refers to the ratio between the specific components in a sample. It can characterize the respective chemical compositions of different samples and is used to determine whether the sources of the two samples are the same (Liu et al., 2021). Because it is less affected by the outside world and is a more diversified evaluation criterion, it is widely used in the traceability and identification of pollutants in the environment (Biache et al., 2014), medical disease diagnosis (Nauck and Meier, 2012), and identification of Chinese medicinal materials in food items (Wu et al., 2022). It is also used to monitor the quality related to the production process of Chinese medicinal materials, about which there are relatively few reports. For example, in Traditional Chinese Medicine, the head, body, and tail of the *A. sinensis* roots are used to treat different diseases owing to their different pharmaceutical functions (Yang et al., 2021). A large amount of research evidence suggests that the difference in the amount of the components in the head, body, and tail of the roots may be the main reason for its different pharmaceutical efficacies (Xue et al., 2012). Thus, it may be a more comprehensive strategy to evaluate the quality of medicinal materials using multiple methods of multi-component monitoring and characteristic-component diagnostic ratios. During the planting process, the early flowering rate of *A. sinensis* reaches 20–30%. Once early flowering occurs, the roots of *A. sinensis* are lignified and cannot be used as medicine, which has resulted in a huge waste of resources and economic loss. The ratios of ligustilide/butylphthalide (1885 in ZT, 37 in ZC) and ligustilide/senkyunolide A (814 in ZT, 12 in ZC) also revealed a steady-state imbalance when plants are flowering early. Therefore, cultivating early flowering-resistant plants has become more popular, resulting in more effective plant ingredients available for medicinal use.

This study aimed to determine the key enzymes in phthalide accumulation and the molecular mechanism underlying the synthesis and accumulation of phthalide components to provide a feasible strategy for increasing the phthalide levels in the flowering *A. sinensis* to reduce waste, improve the market supply of medicinal raw materials, and alleviate the economic losses of farmers. Studies on the mechanism underlying phthalide biosynthesis have been reported previously, but the enzymes related to the accumulation of phthalides, especially the enzymes involved in different phthalide transformations, have not yet been identified, requiring further investigation (León et al., 2017). In this study, we combined transcriptome and targeted metabolite profile analyses to explore potential enzymes or pathways involved in the differential regulation of phthalide accumulation. Six enzymes, including phospho-2-dehydro-3-deoxyheptonate aldolase 2 (17), shikimate dehydrogenase (21), primary amine oxidase-like (23, 24), polyphenol oxidase (36), tyrosine decarboxylase (38), and shikimate *O*-hydroxycinnamoyl transferase (43), have shown potential for the regulation of phthalide accumulation.

Phospho-2-dehydro-3-deoxyheptonate aldolase 2 (17) and shikimate dehydrogenase (21) catalyze the first and fourth committed steps of the shikimate pathway, respectively, both of which are required for the synthesis of aromatic amino acids and other aromatic metabolites in bacteria, microbial eukaryotes, and plants (Bagautdinov and Kunishima, 2007; Heyes et al., 2014). Polyphenol oxidase (36) is a group of Cu-containing enzymes that catalyzes the oxidation of several phenols to *o*-quinones (Prexler et al., 2019). Polyphenol oxidases participate in two oxidation reactions. The first is hydroxylation of the ortho-position adjacent to an existing hydroxyl group. The second mechanism is the oxidation of o-dihydroxybenzenes to o-benzoquinones (Taranto et al., 2017). Tyrosine decarboxylase (38), a pyridoxal phosphate-dependent amino acid decarboxylase, is a key enzyme in dopamine synthesis, and its catalytic products are implicated in the defense response (Gayathri and Manoj, 2020). In the tyrosine metabolic pathway, l-tyrosine is used as a substrate to catalyze its decarboxylation to form tyramine. Moreover, tyrosine decarboxylase can also decarboxylate phenylalanine to produce phenylethylamine, another biogenic amine (Marcobal et al., 2012). Shikimate *O*-hydroxycinnamoyl transferase (43) catalyzes the synthesis of shikimate and quinate esters. It appears to control the biosynthesis and turnover of major plant phenolic compounds, such as lignin and chlorogenic acid (Hoffmann et al., 2003). Although the specific reactions that participate in the synthesis and transformation of phthalides are unknown, the catalytic reaction of heterologously expressed proteins in *E. coli* has proved that they promote the accumulation of certain phthalides.

**Figure 6:**
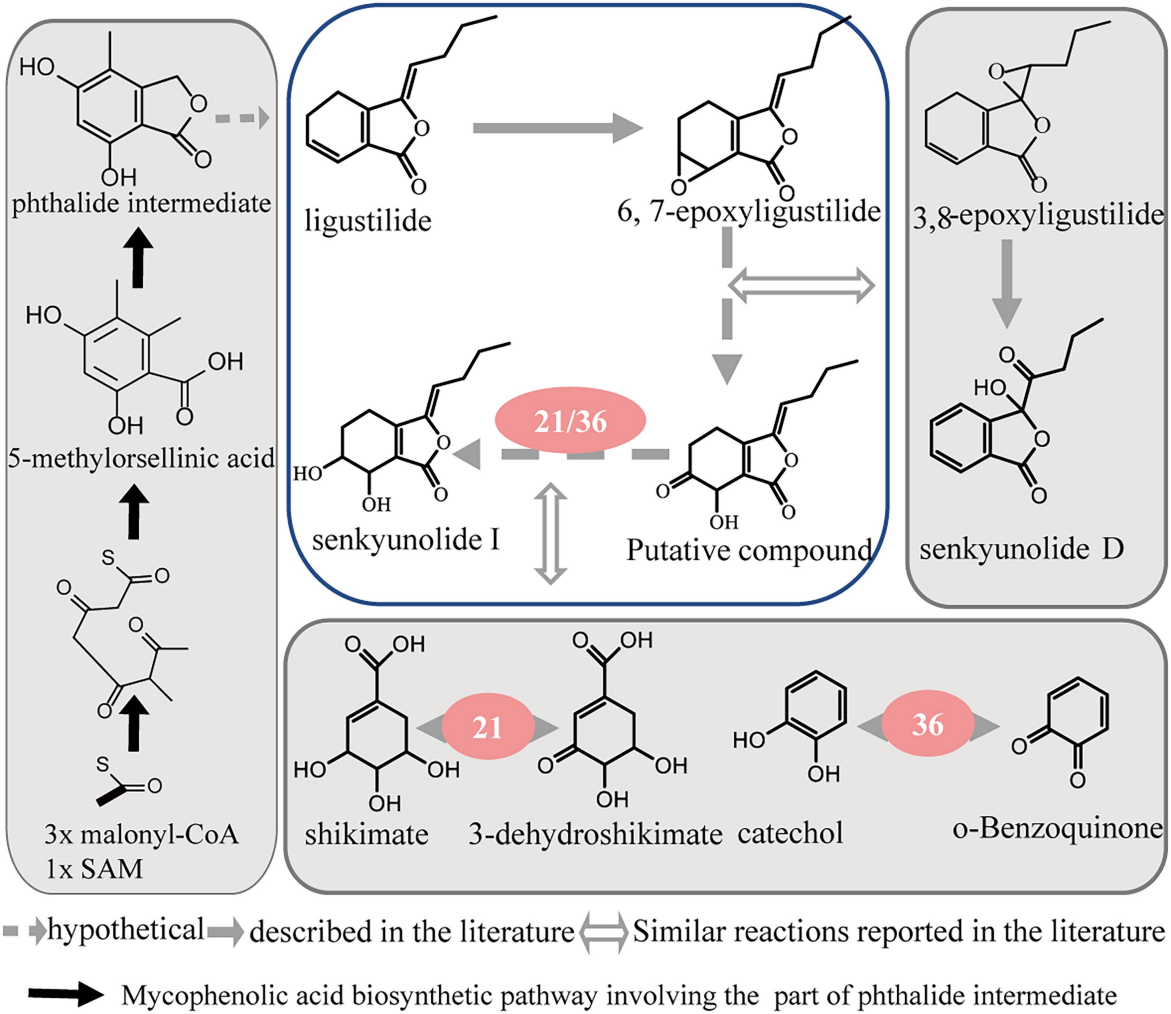
Proposed reaction pathways from ligustilide to senkyunolide I.

Through the analysis of these enzyme reactions, it is speculated that they may regulate the conversion of different phthalide components through various pathways, such as oxidation, isomerization, and hydroxylation. As the most abundant phthalide in *A. sinensis*, ligustilide is a volatile and unstable compound with an α, β-unsaturated lactone in its structure. Senkyunolide I and 6, 7-epoxyligustilide were the major degradation products when ligustilide was stored at room temperature under direct sunlight. Ligustilide is likely to degrade into 6, 7-epoxyligustilide through oxidation and then transform into senkyunolide I by further hydrolysis (Zou et al., 2013). In addition, senkyunolide I and 6, 7-epoxyligustilide are also the *in vivo* metabolites of ligustilide (Ding et al., 2008; Yan et al., 2008). These studies suggest that they may have the same synthesis and transformation pathways in plants. In our study, polyphenol oxidase (36) and shikimate dehydrogenase (21) contributed to the transformation of ligustilide to senkyunolide I. Polyphenol oxidase (36) can react with o-benzoquinone and water to produce catechol and oxygen. 3-dehydroshikimate, NADPH, and H^+^ generate shikimate and NADP^+^ under the action of shikimate dehydrogenase (21). In the above reaction, polyphenol oxidase (36) and shikimate dehydrogenase (21) mainly act on the conversion of the compound from a carbonyl to a hydroxyl group. The proposed reaction pathways from ligustilide to senkyunolide I are shown in Figure 6 by summarizing the enzyme function and the present study on phthalide transformation (Schinkovitz et al., 2008).

Overall, our study explored candidate enzymes corresponding to different phthalide compounds that attempt to build phthalide biosynthetic and transformed pathways. Seven candidate isoforms involved in phthalide accumulation and transformation have been identified. Due to the lack of relevant research, we could not fully clarify the mechanism of action of each enzyme. Only the proposed reaction pathway from ligustilide to senkyunolide I is shown. Further investigations into the enzymatic properties and their regulatory mechanisms are required to completely understand phthalide accumulation and transformation in *A. sinensis.* These findings may also provide insights into the genes that can make for potential genetic engineering targets for enriching phthalides in *A. sinensis*.

## Data Availability Statement

The datasets presented in this study can be found in online repositories. The names of the repository/repositories and accession number(s) can be found at: https://www.ncbi.nlm.nih.gov/, PRJNA749925.

## Author Contributions

W-MF, PL, HY, and J-AD conceived and designed the experiments. W-MF performed the experiments. W-MF, PL, E-XS, and SZ analyzed the data. J-AD, D-WQ, SZ, GY, HY, and SJ contributed reagents, materials, and analysis tools. W-MF and PL wrote the manuscript. All authors have read and approved the final manuscript.

## Funding

This research was supported financially by Innovation Team and Talents Cultivation Program of National Administration of Traditional Chinese Medicine (ZYYCXTD-D-202005). China Agriculture Research System of MOF and MARA (No. CARS-21), Ministry of Finance Central Level of the Special (No. 2060302), National Natural Science Foundation of China (81773848). This work was also partly sponsored by Six talents peaks project in Jiangsu Province (JNHB-066), Jiangsu Province 333 High-level Talents Training Project, Qing Lan Project, the Major Projects of Natural Science Foundation of Universities in Jiangsu Province (19KJA320002) and Natural Science Foundation of Jiangsu Province, China (BK20201403).

## Conflict of Interest

The authors declare that the research was conducted in the absence of any commercial or financial relationships that could be construed as a potential conflict of interest.

## Publisher’s Note

All claims expressed in this article are solely those of the authors and do not necessarily represent those of their affiliated organizations, or those of the publisher, the editors and the reviewers. Any product that may be evaluated in this article, or claim that may be made by its manufacturer, is not guaranteed or endorsed by the publisher.

## Footnotes

^1^ https://www.ncbi.nlm.nih.gov/bioproject/

## Abbreviations

UHPLC-MS/MS: ultra-high performance liquid chromatography-tandem mass spectrometry
qRT-PCR: quantitative real-time polymerase chain reaction
MeJA: methyl jasmonate
GO: Gene Ontology
KEGG: Kyoto Encyclopedia of Genes and Genomes
MRM: multiple reaction monitoring
FPKM: fragments per kilobase of exon per million fragments mapped
COG: Clusters of Orthologous Genes
KOG: EuKaryotic Orthologous Groups
