# Supplementary material for: Investigation of Enzymes in the Phthalide Biosynthetic Pathway in Angelica sinensis Using Integrative Metabolite Profiles and Transcriptome Analysis
Source: Front Plant Sci. 2022 Jul 1;13:928760. doi: 10.3389/fpls.2022.928760 (PMC9286521; doi:10.3389/fpls.2022.928760)
Supplement: Supplementary file 1 [file Data_Sheet_1.docx]

**Table S1** Gene specific primer pairs used in qRT-PCR analysis

| **Isoform** | **Gene** |  | **Sequence(5’-3’)** | **Tm**  **℃** | **GC Content** | **Product size** |
| --- | --- | --- | --- | --- | --- | --- |
| c73776/f3p0/2391_YS | 1 | forward | AAGTGATGGTGACGGTGATAG | 59.5 | 48% | 228 |
|  |  | reverse | CCAAAGAATTTCCAACCAGTGG | 60.1 | 45% |  |
| c25107/f2p0/1677_YS | 2 | forward | GGTCTCGCTCCAAAGTTTGG | 60.5 | 55% | 198 |
|  |  | reverse | CTGTCCACCAGATGTGTAGTG | 61.2 | 52% |  |
| c66845/f2p0/2186_YS | 3 | forward | GAGGGTACTTCGATAACTATGG | 60.1 | 45% | 222 |
|  |  | reverse | ACGCAGGGTAATTGACACCTC | 61.2 | 52% |  |
| c86518/f3p3/1525_ZPZC | 4 | forward | TGGTGCAGAACTTGTTGGTAC | 59.5 | 48% | 208 |
|  |  | reverse | GGTTTCTCCATAGCAACGAGG | 61.2 | 52% |  |
| c24427/f1p3/1899_ZPZC | 5 | forward | CCGGAGATTCTATTGTAGCTG | 59.5 | 48% | 165 |
|  |  | reverse | GTGACTGCAAGAAACCCTGG | 60.5 | 55% |  |
| c33491/f1p0/2070_ZPZT | 6 | forward | TGTCACAACTCCAAAAGTCGG | 59.5 | 48% | 204 |
|  |  | reverse | GAACTATGTTACACACTGCCGA | 60.1 | 45% |  |
| c1490/f3p2/2316_ZPZC | 7 | forward | CCAAGGGACTTTGATCAGCC | 60.5 | 55% | 157 |
|  |  | reverse | TGCTGAAGAAAGGGTCGCC | 59.5 | 58% |  |
| c48858/f1p0/1024_ZP | 8 | forward | CTCCGGCGGCTCATTATTC | 59.5 | 58% | 156 |
|  |  | reverse | TGCAGCGGTAGAGTCAACGT | 60.5 | 55% |  |
| c107121/f1p0/1718_ZPZC | 9 | forward | CAAGAATCGGTCTCGCTGG | 59.5 | 58% | 183 |
|  |  | reverse | TCACGAACGCCTCAGGATC | 59.5 | 58% |  |
| c36451/f1p1/1280_ZPZC | 10 | forward | CAGTCGGGAAGCTCTCGAA | 59.5 | 58% | 194 |
|  |  | reverse | CTTGCTGCACCGTCCATTTAC | 61.2 | 52% |  |
| c13963/f1p1/1531_YS | 11 | forward | ACCACCCATTTCTAGACTCTG | 59.5 | 48% | 200 |
|  |  | reverse | GGAAATCAAATGGAGGGTGTC | 59.5 | 48% |  |
| c42564/f1p2/1350_ZPZC | 12 | forward | AAGGTGGGAAGAATGGCTGG | 60.5 | 55% | 190 |
|  |  | reverse | GAAGGTTAAGTGTTGCTGCAG | 59.5 | 48% |  |
| c22794/f1p0/1116_ZP | 13 | forward | GACTGAGTGCATTGGTGGATC | 61.2 | 52% | 210 |
|  |  | reverse | TCTTAAGGCCCAGCTCAACC | 60.5 | 55% |  |
| c28447/f1p2/1921_ZPZC | 14-1 | forward | CTATTTCAGCTGTCCATTCTCC | 60.1 | 45% | 206 |
|  |  | reverse | GTCAGGGTACTCAGGAAGCT | 60.5 | 55% |  |
| c28447/f1p2/1921_ZPZC | 14-2 | forward | AGGAGAGTGAACAGGGTGACA | 61.2 | 52% | 190 |
|  |  | reverse | AGGCCAGATGTAGAATCCAATC | 60.1 | 45% |  |
| c114409/f1p0/1841_YS | 17-1 | forward | CACTCAATATCCCCTCTCCAC | 61.2 | 52% | 151 |
|  |  | reverse | TCCATTTCACAGGGGGTGTTG | 61.2 | 52% |  |
| c114409/f1p0/1841_YS | 17-2 | forward | GAATGGCTGGTCAGTTTGCC | 59.5 | 48% | 144 |
|  |  | reverse | GTTGCTGCAGCTTGAGTATAAG | 60.5 | 55% |  |
| c27078/f1p2/1676_ZPZC | 20 | forward | CACGGAAAGAGAGTTCTTGTG | 59.5 | 48% | 180 |
|  |  | reverse | CTCAATTGCCTTGTCGTAGAC | 59.5 | 48% |  |
| c82794/f1p0/1954_ZPZT | 21 | forward | CAAGTGGTGCTGACATAGTGA | 60.5 | 55% | 102 |
|  |  | reverse | TCGGGCAAAGTATCCGTGAC | 60.5 | 55% |  |
| c98537/f1p0/1126_ZPZT | 23 | forward | CATCACGGGCATGGAGATTC | 59.5 | 48% | 117 |
|  |  | reverse | CAAGATTCCACTAAGTCCAACC | 59.5 | 48% |  |
| c10933/f1p0/1354_ZP | 24 | forward | TTGCTGCAGATGGAAGCCCA | 59.5 | 48% | 127 |
|  |  | reverse | CATGCGAGCAACTAGAGTCAC | 59.5 | 48% |  |
| c41582/f3p0/2370_YS | 25 | forward | CTTTATCTCTTGACGAGCCTG | 59.5 | 48% | 197 |
|  |  | reverse | ATCATCCTAGGTCACAACAGG | 59.5 | 48% |  |
| c172739/f1p0/1655_YS | 27-1 | forward | GAATCGCAGACTTGCTAAACC | 59.5 | 48% | 158 |
|  |  | reverse | GTAGAGGAATAAGTTTGCGGTC | 60.1 | 45% |  |
| c172739/f1p0/1655_YS | 27-2 | forward | ACTTCACAAATTTCACTGGCGG | 60.1 | 45% | 175 |
|  |  | reverse | CCATACTCATAATTCGCCACAG | 60.1 | 45% |  |
| c49867/f1p0/1551_ZPZC | 30 | forward | AACCCAGGAAATCCTTGTGGA | 59.5 | 48% | 234 |
|  |  | reverse | AACAAGCCACCCAAGTCTCC | 60.5 | 55% |  |
| c49867/f1p0/1551_ZPZC | 32 | forward | ACTACTGCAGGGTCATATGCT | 59.5 | 48% | 188 |
|  |  | reverse | ACATATGGGTTCACAACGTGG | 59.5 | 48% |  |
| c1835/f4p0/1736_ZP | 33 | forward | TCTGGTACTGGCTCACTGAG | 60.5 | 55% | 195 |
|  |  | reverse | AAGATCCTCTAGCATGCCTAC | 59.5 | 48% |  |
| c61565/f1p0/1035_YS | 34 | forward | GTACCTGGGTGGAGACTTG | 59.5 | 58% | 155 |
|  |  | reverse | GTGTCTCCAAGGATCTGAGG | 60.5 | 55% |  |
| c24590/f1p0/1723_ZPZC | 35 | forward | CAGGACAGATATGCTCTAAGG | 59.5 | 48% | 195 |
|  |  | reverse | TGTATTGTCCATCGACACTCC | 59.5 | 48% |  |
| c49687/f1p0/1149_ZPZC | 36 | forward | GATCGAATGTGGAGCGTATGG | 59.5 | 48% | 130 |
|  |  | reverse | CATCTTGGTATACATATCCGAG | 58.4 | 41% |  |
| c2460/f2p1/1076_ZPZC | 37 | forward | CCGCAAAGATATCGAGGATCC | 61.2 | 52% | 147 |
|  |  | reverse | AGCCACGGAAGAGGAACATC | 60.5 | 55% |  |
| c12383/f1p0/1141_ZP | 38 | forward | CAGAGTTCCGCCATTACCTTG | 61.2 | 52% | 237 |
|  |  | reverse | TCAATGACCTGAACCGTCTTC | 59.5 | 48% |  |
| c26825/f1p0/1628_ZPZT | 38.1 | forward | TGACCAAACTCATTCTGCCCT | 60.5 | 55% | 124 |
|  |  | reverse | TCGCTTAGGACAGAGCTGG | 60.1 | 45% |  |
| c2791/f1p0/1420_ZPZT | 38.2 | forward | CCTCAAATTTCCAGCTCTGTC | 59.5 | 48% | 132 |
|  |  | reverse | CTGGCAATGGATCAACTGTGG | 61.2 | 52% |  |
| c349510/f1p0/975_YS | 40 | forward | GAGAGTGCCCTGGAGTTGTT | 60.5 | 55% | 155 |
|  |  | reverse | GTCAGGGAGGTATTGCTCTAG | 61.2 | 52% |  |
| c53819/f1p0/1923_ZPZT | 41 | forward | GGCGAATCCGTTCTTCACCT | 60.5 | 55% | 164 |
|  |  | reverse | GTAAACAATCCTGAGGAGCATC | 60.1 | 45% |  |
| c73461/f1p0/1689_ZPZT | 42 | forward | GTGTGGTGGACTTGCTATATG | 59.5 | 48% | 157 |
|  |  | reverse | AGATGCGAAATCAAAGCCAACC | 60.1 | 45% |  |
| c113652/f1p0/1125_YS | 43 | forward | AGGATGTGGGGTGCACCATA | 60.5 | 55% | 157 |
|  |  | reverse | ATCAAACACTGGAGTAGGTGG | 59.5 | 48% |  |
| c70277/f1p0/1551_ZPZC | 44 | forward | ATGCTCATGTCAAGGGCGAC | 60.5 | 55% | 168 |
|  |  | reverse | AAGCATCGCCAAGTTTGTGTG | 59.5 | 48% |  |
| c23271/f1p0/1511_ZPZT | 45 | forward | AGCTCTTCAGGATCCTACGTT | 59.5 | 48% | 156 |
|  |  | reverse | CACATTACCCCATATTGAGCC | 59.5 | 48% |  |
| c9126/f1p0/1016_ZP | 46 | forward | TGACTCAATGGTTGCAGACCA | 59.5 | 48% | 155 |
|  |  | reverse | CTACAACGCCTACCTTAGTAC | 59.5 | 48% |  |
| c16304/f2p0/1341_ZP | 47 | forward | CCACAGTAGTCGGAGAAGATG | 61.2 | 52% | 182 |
|  |  | reverse | TGGAACTCCTCCCTCAAGCA | 60.5 | 55% |  |
| c11197/f1p0/1188_ZP | 49 | forward | AGTGACGGGCACAGTGATCA | 60.5 | 55% | 156 |
|  |  | reverse | CAATGGCACAATGAAACTCCAC | 60.1 | 45% |  |
| c10154/f1p0/1513_ZP | 50 | forward | GGCACAATGAAACTCCACTTAC | 60.1 | 45% | 154 |
|  |  | reverse | TGAGTGACGGGCACAGTGAT | 60.5 | 55% |  |
| c2178/f5p0/1771_ZPZC | 51 | forward | CCTTATCGGCCACCAAACTTG | 61.2 | 52% | 182 |
|  |  | reverse | GGTGGTACACACTACCGTG | 59.5 | 58% |  |
| c20355/f1p1/1848_ZPZC | 52-1 | forward | CCTTACCCACAGCCACTCTT | 60.5 | 55% | 213 |
|  |  | reverse | CAAAGAGAGTAGACCCAGGAG | 61.2 | 52% |  |
| c20355/f1p1/1848_ZPZC | 52-2 | forward | ACCTACGCATTGTGGAGCATT | 59.5 | 48% | 248 |
|  |  | reverse | TCGCAATTCTAGTGCCTTCCAT | 60.1 | 45% |  |
| c40544/f2p3/1238_ZPZC | 54 | forward | CCATTGGGGGTTTCGGTTCT | 60.5 | 55% | 184 |
|  |  | reverse | AGCAGGGACAAGACAGTTCC | 60.5 | 55% |  |
| c42460/f1p0/1354_ZPZC | 57 | forward | AGCAGGGACAAGACAGTTCC | 60.5 | 55% | 120 |
|  |  | reverse | AGGCCTTCACAAGCCAATCC | 60.5 | 55% |  |
| c3114/f1p0/2683_YS | 58 | forward | GACGGCTGGACAAGCTTATG | 60.5 | 55% | 168 |
|  |  | reverse | CTTGAGTGTAGCCTTGTTAGTG | 60.1 | 45% |  |
| c3114/f1p0/2683_YS | 60-1 | forward | CCATGACCACCACTGACACA | 61.2 | 52% | 176 |
|  |  | reverse | TGCCACCAGAGGGATGTTATA | 59.5 | 48% |  |
| c3114/f1p0/2683_YS | 60-2 | forward | GGAGGCTGGCTAATCTTGGTA | 61.2 | 52% | 152 |
|  |  | reverse | GGCACCTCTATAATCTACCTC | 59.5 | 48% |  |
| c868/f6p0/1699_ZPZC | 63 | forward | AGCGGTTAGAGGATATGGAAG | 59.5 | 48% | 180 |
|  |  | reverse | TAGAAACCCAGGGGCATGTAG | 61.2 | 52% |  |
| c46788/f1p2/1649_ZPZC | 64 | forward | GGAAAGAAGCAATTTGAGGCCA | 60.1 | 45% | 143 |
|  |  | reverse | CAGACCTTTGATACCCATGGA | 59.5 | 48% |  |
| c223029/f1p0/1208_YS | 65 | forward | GTGGGTGGTGGTTGTGATG | 59.5 | 58% | 161 |
|  |  | reverse | TACGTAGTCTTATCAGCCACC | 59.5 | 48% |  |
| c270786/f15p1/1176_YS | 66 | forward | CTCATCAGACGACGCTGTAAC | 61.2 | 52% | 184 |
|  |  | reverse | TAACTCGAAACCGAACGGCAC | 61.2 | 52% |  |
| c208236/f3p0/1304_YS | 67 | forward | GATCGATTCGGTCCATATCACT | 60.1 | 45% | 178 |
|  |  | reverse | TGCAAGGAGGGAGTAACGCA | 60.5 | 55% |  |
| c42049/f1p0/1464_YS | 68 | forward | CTGGAGGTAAGCTGAATCGAG | 61.2 | 52% | 208 |
|  |  | reverse | ACCTTGGGCACTCTGAACC | 59.5 | 58% |  |
| c2712/f1p4/1371_ZPZC | 69 | forward | AACGAACCAAGCACATACTGG | 59.5 | 48% | 143 |
|  |  | reverse | TCAGCAACCTCATCGGGGT | 59.5 | 58% |  |
| c32731/f1p4/1164_ZPZC | 70 | forward | TCCTTCAGCAACGTTCTGCAA | 59.5 | 48% | 164 |
|  |  | reverse | AGCAGGAATACCGAGCTCATC | 61.2 | 52% |  |
| c84658/f1p2/1193_ZPZC | 73 | forward | GATGTTGCTGACCCTTCAAGT | 59.5 | 45% | 212 |
|  |  | reverse | ACGCATTGTTCAGTTAACTCGT | 58.4 | 41% |  |
| c13151/f4p0/1872_ZP | 75 | forward | TCCGCAGGATGGCAGTTATG | 60.6 | 55% | 183 |
|  |  | reverse | TCGCAACCACCATAGTATCTG | 59.5 | 48% |  |
| c12244/f1p0/1638_ZPZC | 78 | forward | GAGAGCATGGATTCTTCCTTG | 59.5 | 48% | 196 |
|  |  | reverse | GTTTCCTTCCACGGCAACTTG | 61.2 | 52% |  |
| c4898/f1p1/951_ZPZC | 79 | forward | GATCCAAGCTCGATAGTTGAG | 59.5 | 48% | 210 |
|  |  | reverse | TGACACCAGGCCGCATACTT | 60.5 | 55% |  |
| c174559/f1p0/2150_YS | 80 | forward | GGTCGAATGCTTGCAAGAGTA | 59.5 | 48% | 200 |
|  |  | reverse | AGCCCTCCAACTTCATCTTGAT | 60.1 | 48% |  |
| c70349/f1p1/1158_ZPZC | 81 | forward | AAGGGACGTGATGGGAAGG | 59.9 | 58% | 204 |
|  |  | reverse | TCACTGTCGGTGTGTGGGA | 59.9 | 58% |  |
| c12185/f1p1/1200_ZPZC | 82 | forward | GTAGCTTTCGGATTGTAATGCA | 58.4 | 41% | 197 |
|  |  | reverse | ACGACACCGACAGCTAGGTT | 60.5 | 55% |  |
| c22261/f1p1/1664_ZPZC | 83 | forward | AATGCGAGCCACCACTCATG | 60.5 | 55% | 201 |
|  |  | reverse | TACCCCATTCTCTTGATGCTTC | 60.1 | 45% |  |
| c9442/f1p0/1340_ZP | 84 | forward | AAGATACTTGTGAGGTCTCGCT | 60.2 | 45% | 202 |
|  |  | reverse | AGCATTTTCATAGGCAGAAGCT | 58.4 | 42% |  |
| c12930/f1p0/1380_ZP | 85 | forward | CATGGCATCCCTGCTGAATTTA | 60.1 | 45% | 153 |
|  |  | reverse | AACCCATCCTACGTCGCCAT | 60.5 | 55% |  |
| c51966/f1p0/1690_ZPZC | 86 | forward | TCGACCCTAATACATTGGACAC | 60.1 | 45% | 198 |
|  |  | reverse | GGCAATTAACCCTACCGATAAC | 60.1 | 45% |  |
| c41588/f5p0/1491_ZP | 87 | forward | ATTTGGCACTGCCCATCGAG | 60.5 | 55% | 156 |
|  |  | reverse | GAACCGCCAACAATTGCAGCA | 61.2 | 52% |  |
| c26405/f1p0/1686_ZPZT | 90 | forward | TTCTCACCTGGGACGACCAA | 60.5 | 55% | 199 |
|  |  | reverse | CTCTTCCTCCTTATAGAATCTC | 58.4 | 41% |  |
| c86745/f1p0/821_YS | 91 | forward | AGCGATATGGTCGGGCACA | 59.5 | 58% | 172 |
|  |  | reverse | CTCCCTTCTTGTTTCTCTTCAC | 60.1 | 45% |  |
| c112201/f1p0/1591_YS | 92 | forward | GTGTTTATGGTGGGAAAGGTGT | 60.1 | 45% | 201 |
|  |  | reverse | CTGCACCAGCTCTACATACACT | 62.1 | 52% |  |
| c28038/f1p5/1383_ZPZC | 93 | forward | GGCTAGTGTCCTGAACATTCC | 61.2 | 52% | 205 |
|  |  | reverse | GTGGTATACTTCAACTCCCATC | 60.1 | 45% |  |
| c26756/f1p1/1197_YS | 98 | forward | AGGATGCCTGCAACGTTGGA | 60.5 | 55% | 148 |
|  |  | reverse | AATTCTGAGGCTGCAACATCC | 59.5 | 48% |  |
| c40133/f2p2/1467_ZPZC | 99 | forward | GAGGTAGCCATGCTGGAAAC | 60.5 | 55% | 168 |
|  |  | reverse | CTTCATCTCCAACGTTGCAGG | 61.2 | 52% |  |
| c270956/f26p0/1681_YS | 100 | forward | TGATTGGCAAGGACCCCACT | 60.5 | 55% | 171 |
|  |  | reverse | GAGGAATGTTCAGGACACTAG | 59.5 | 48% |  |
| c10292/f1p1/1652_ZP | 101 | forward | CCTCTACAGCTCTACTCGTATT | 60.1 | 45% | 217 |
|  |  | reverse | TCAGTAGCAGGTAGAGATGCCT | 62.1 | 50% |  |
| c48717/f1p1/1953_ZPZC | 103 | forward | CCGCTTCTCGATCTGTACCTAT | 62.1 | 50% | 185 |
|  |  | reverse | TCGAATCTCAGGTCCCTTGG | 60.5 | 55% |  |
| c52629/f1p3/3108_ZPZC | 104 | forward | GATAACAGAGCATGTCCTTCC | 59.5 | 48% | 188 |
|  |  | reverse | ATTGCGGCCCAACAAGTGGT | 60.5 | 55% |  |
| c25635/f1p1/1013_ZP | 105 | forward | GTTGCTCGTGGTGATCTTGG | 60.5 | 55% | 167 |
|  |  | reverse | TCAGTTGCCTCAGCACGAGT | 60.5 | 55% |  |
| c12117/f1p0/2093_ZPZC | 107 | forward | TTCTCCCAACGGTCCCCTCA | 62.5 | 60% | 152 |
|  |  | reverse | CACGTGAACTTGTAGAAGGACC | 62.1 | 50% |  |
| c20820/f2p0/1943_ZP | 108 | forward | CCTGACGAGGACAAGGATG | 59.5 | 58% | 152 |
|  |  | reverse | ACCTCCGTAACCTCTAGCCA | 60.5 | 55% |  |
| c12685/f2p0/1154_ZPZC | 109 | forward | CAGAATCCGGGTGAGTCAC | 59.5 | 58% | 144 |
|  |  | reverse | AGCAACATAAGTCAGCACATCC | 60.1 | 45% |  |
| c120201/f1p0/1165_YS | 111 | forward | GATGGCTAATGAAACAGGCCT | 59.5 | 48% | 144 |
|  |  | reverse | CACGTTGGCCTGTTAGGATG | 60.5 | 55% |  |
| c3592/f1p0/2969_ZPZC | 112 | forward | CAGAACCCAGTATTCCCAGAC | 61.2 | 52% | 122 |
|  |  | reverse | CTCATTCATACCCAACTCTGG | 59.5 | 48% |  |
| CL591.Contig1_DGT | 18sRNA | forward | CTATGTCTGGACCTGGTAAG | 58.4 | 50% | 163 |
|  |  | reverse | AGGACTCCACTGGCACCT | 58.4 | 61% |  |

**Table S2** Content levels of the tested components across samples. (n=6)

| samples | Ligustilide *(mg/g) | Butylphthalide *(µg/g) | butylidenephthalide (µg/g) | senkyunolide H *(µg/g) | senkyunolide I (µg/g) | senyunolide A *(µg/g) | Sum *(mg/g) |
| --- | --- | --- | --- | --- | --- | --- | --- |
| ZT-1 | 5.883 | 4.790 | 45.82 | 2.279 | 53.58 | 4.968 | 5.995 |
| ZT-2 | 5.985 | 3.186 | 44.34 | 1.696 | 58.34 | 4.936 | 6.097 |
| ZT-3 | 6.586 | 4.488 | 54.81 | 1.566 | 65.48 | 6.102 | 6.718 |
| ZT-4 | 4.651 | 2.284 | 29.07 | 1.613 | 30.73 | 9.575 | 4.724 |
| ZT-5 | 4.186 | 1.931 | 21.21 | 1.239 | 26.30 | 8.636 | 4.246 |
| ZT-6 | 4.676 | 1.848 | 27.18 | 0.7012 | 26.63 | 10.75 | 4.743 |
| ‾x±SD | 5.33±0.95 | 3.09±1.30 | 37.07±13.10 | 1.52±0.52 | 43.51±17.60 | 7.49±2.49 | 5.42±0.98 |
| ZC-1 | 6.541 | 137.5 | 44.90 | 2.040 | 49.12 | 431.1 | 7.205 |
| ZC-2 | 6.713 | 154.5 | 46.65 | 3.039 | 46.29 | 474.9 | 7.439 |
| ZC-3 | 6.644 | 153.6 | 52.97 | 2.040 | 51.78 | 486.1 | 7.391 |
| ZC-4 | 6.266 | 222.8 | 36.57 | 3.100 | 67.76 | 612.0 | 7.208 |
| ZC-5 | 6.281 | 206.9 | 36.07 | 2.806 | 59.17 | 588.8 | 7.175 |
| ZC-6 | 6.191 | 214.4 | 37.64 | 3.746 | 63.06 | 620.2 | 7.130 |
| ‾x±SD | 6.44±0.22 | 181.62±37.10 | 42.47±6.82 | 2.80±0.66 | 56.20±8.46 | 535.51±81.10 | 7.26±0.13 |

* P<0.05 by t test between ZT vs ZC

**Table S3** The MRM transitions and parameters applied of six components

| Name | Formula | RT min | m/z precursor ion | m/z product ion | DP | EP | CE | CXP | Ion mode |
| --- | --- | --- | --- | --- | --- | --- | --- | --- | --- |
| senkyunolide A* | C_12_H_16_O_2_ | 7.51 | 193.027 | 136.8 | 86 | 10 | 17 | 16 | + |
| senkyunolide A | C_12_H_16_O_2_ | 7.51 | 193.027 | 91.0 | 86 | 10 | 27 | 10 | + |
| butylphthalide* | C_12_H_14_O_2_ | 7.87 | 191.058 | 173.0 | 56 | 10 | 13 | 18 | + |
| butylphthalide | C_12_H_14_O_2_ | 7.87 | 191.058 | 144.9 | 56 | 10 | 21 | 16 | + |
| butylidenephthalide* | C_12_H_12_O_2_ | 9.99 | 189.021 | 171.0 | 86 | 10 | 19 | 20 | + |
| butylidenephthalide | C_12_H_12_O_2_ | 9.99 | 189.021 | 127.9 | 86 | 10 | 33 | 14 | + |
| senkyunolide I | C_12_H_16_O_4_ | 4.01 | 225.133 | 207.0 | 91 | 10 | 9 | 24 | + |
| senkyunolide I* | C_12_H_16_O_4_ | 4.01 | 225.133 | 165.0 | 91 | 10 | 23 | 18 | + |
| senkyunolide H | C_12_H_16_O_4_ | 4.19 | 225.121 | 207.0 | 91 | 10 | 9 | 24 | + |
| senkyunolide H* | C_12_H_16_O_4_ | 4.19 | 225.121 | 189.1 | 91 | 10 | 25 | 20 | + |
| ligustilide | C_12_H_14_O_2_ | 9.72 | 191.049 | 115.0 | 126 | 10 | 47 | 50 | + |
| ligustilide* | C_12_H_14_O_2_ | 9.72 | 191.049 | 91.0 | 126 | 10 | 31 | 40 | + |

* The quantification ion

**Table S4** Overview of the transcriptome sequencing dataset and quality check

| sample | clean base(bp) | length | Q20(%) | Q30(%) | GC(%) |
| --- | --- | --- | --- | --- | --- |
| ZC-1 | 7010838300 | 150 | 97.4 | 92.3 | 42.7 |
| ZC-2 | 7151204100 | 150 | 97.5 | 92.6 | 42.4 |
| ZC-3 | 6497895000 | 150 | 97.9 | 93.4 | 42.4 |
| ZT-1 | 6570018600 | 150 | 97.3 | 91.9 | 42.4 |
| ZT-2 | 7377909600 | 150 | 97.7 | 92.8 | 42.4 |
| ZT-3 | 6748411800 | 150 | 97.4 | 92.1 | 42.4 |

**Table S5** The expression of transcript isoforms and phthalides content for correlation analysis in ZC and ZT sample

|  | ZT1 | ZT2 | ZT3 | ZC1 | ZC2 | ZC3 |
| --- | --- | --- | --- | --- | --- | --- |
| ligustilide (mg/g) | 5.88 | 5.62 | 4.43 | 6.54 | 6.45 | 6.24 |
| senkyunolide H (µg/g) | 2.28 | 1.59 | 0.97 | 2.04 | 2.57 | 3.28 |
| senkyunolide I (µg/g) | 53.58 | 48.10 | 26.47 | 49.12 | 59.77 | 61.12 |
| butylidenephthalide (µg/g) | 45.82 | 41.94 | 24.19 | 44.90 | 44.77 | 36.86 |
| butylphthalide (µg/g) | 4.79 | 3.39 | 1.89 | 137.48 | 188.21 | 210.67 |
| senyunolide A (µg/g) | 4.97 | 7.84 | 9.69 | 431.10 | 549.04 | 604.51 |
| c73776/f3p0/2391_YS | 6.83 | 4.33 | 3.58 | 19.31 | 21.34 | 18.56 |
| c25107/f2p0/1677_YS | 8.69 | 9.62 | 7.13 | 25.37 | 21.66 | 13.14 |
| c66845/f2p0/2186_YS | 0 | 0 | 0 | 7.27 | 0 | 0 |
| c86518/f3p3/1525_ZPZC | 0 | 0 | 0 | 57.64 | 68.99 | 32.31 |
| c24427/f1p3/1899_ZPZC | 0 | 0 | 0 | 35.72 | 27.74 | 30.74 |
| c33491/f1p0/2070_ZPZT | 6.45 | 5.1 | 5.4 | 57.96 | 31.19 | 25.98 |
| c1490/f3p2/2316_ZPZC | 22.18 | 12.6 | 10.89 | 61.83 | 67.03 | 42.91 |
| c48858/f1p0/1024_ZP | 6.89 | 9.49 | 7.22 | 22.14 | 18.61 | 22.25 |
| c107121/f1p0/1718_ZPZC | 0 | 0 | 0 | 119.57 | 56.11 | 3.42 |
| c36451/f1p1/1280_ZPZC | 0 | 0 | 0 | 0.51 | 0.75 | 0.27 |
| c13963/f1p1/1531_YS | 4.74 | 4.1 | 7.56 | 15.37 | 12.65 | 11.68 |
| c42564/f1p2/1350_ZPZC | 0 | 0 | 0.03 | 18.12 | 0 | 0 |
| c22794/f1p0/1116_ZP | 0 | 0 | 0 | 26.06 | 23.71 | 15.1 |
| c28447/f1p2/1921_ZPZC | 0 | 0 | 0 | 24.21 | 3.09 | 0 |
| c15961/f1p0/995_ZP | 0 | 0 | 0 | 22.87 | 0 | 12.92 |
| c12700/f1p2/2098_ZPZC | 0.51 | 0 | 0.49 | 11.25 | 10.12 | 14.18 |
| c114409/f1p0/1841_YS | 2.88 | 5.33 | 8.86 | 58.47 | 41.5 | 35.71 |
| c40419/f3p2/1688_ZPZC | 8.84 | 10.83 | 11.02 | 56.19 | 34.4 | 39.87 |
| c18236/f1p2/840_ZPZC | 21.87 | 35.26 | 43.2 | 90.9 | 91.72 | 170.05 |
| c27078/f1p2/1676_ZPZC | 0.51 | 0 | 0 | 12.44 | 6.46 | 6.78 |
| c82794/f1p0/1954_ZPZT | 0.4 | 0.37 | 0.6 | 2.32 | 3.28 | 3.12 |
| c98537/f1p0/1126_ZPZT | 0 | 0 | 0 | 0 | 0 | 7.36 |
| c10933/f1p0/1354_ZP | 0.07 | 0.69 | 0 | 2.27 | 8.66 | 5.36 |
| c41582/f3p0/2370_YS | 1.64 | 1.52 | 1.04 | 3.84 | 9.49 | 5.99 |
| c172739/f1p0/1655_YS | 2.71 | 5.22 | 3.7 | 22.62 | 15.97 | 11.84 |
| c24347/f1p0/1514_ZP | 1.18 | 1.06 | 0.54 | 4.17 | 3.35 | 2.38 |
| c16431/f1p0/1343_YS | 2.82 | 2.42 | 5.56 | 11.5 | 11.74 | 10.53 |
| c26825/f1p0/1628_ZPZT | 3.46 | 2.3 | 3.88 | 12.04 | 12.2 | 7.24 |
| c49867/f1p0/1149_ZPZC | 4.85 | 2.4 | 3.39 | 10.34 | 15.41 | 10.9 |
| c61565/f1p0/1035_YS | 4.2 | 4.71 | 5.64 | 11.33 | 17.43 | 7.97 |
| c3664/f3p0/1817_YS | 2.14 | 3.03 | 2.31 | 12.29 | 8.51 | 9.6 |
| c61565/f1p0/1035_YS | 4.2 | 4.71 | 5.64 | 11.33 | 17.43 | 7.97 |
| c24590/f1p0/1723_ZPZC | 0 | 0 | 0 | 0.78 | 0.88 | 1.61 |
| c49687/f1p0/1551_ZPZC | 0 | 0 | 0 | 0 | 0 | 8.04 |
| c2460/f2p1/1076_ZPZC | 1.66 | 3.83 | 9.45 | 82.07 | 31.26 | 41.25 |
| c349510/f1p0/975_YS | 0 | 0 | 0 | 0 | 95.8 | 0 |
| c53819/f1p0/1923_ZPZT | 1.54 | 0.94 | 1.79 | 11.63 | 4.17 | 5.85 |
| c73461/f1p0/1689_ZPZT | 0 | 0 | 0 | 26.71 | 28.78 | 19.82 |
| c113652/f1p0/1125_YS | 0 | 0 | 0 | 14.94 | 15.92 | 7.15 |
| c70277/f1p0/1551_ZPZC | 0 | 0 | 0 | 11.62 | 11.42 | 0 |
| c23271/f1p0/1511_ZPZT | 7.58 | 4.33 | 11.21 | 50.12 | 36.2 | 25.59 |
| c9126/f1p0/1016_ZP | 0 | 0 | 0 | 5.07 | 41.07 | 0 |
| c16304/f2p0/1341_ZP | 2.73 | 3.12 | 8.91 | 29.24 | 34.03 | 17.05 |
| c17892/f1p1/873_ZPZC | 0 | 0 | 0 | 22.5 | 21.84 | 6.76 |
| c11197/f1p0/1188_ZP | 0 | 0 | 0 | 3.66 | 5.97 | 4.51 |
| c10154/f1p0/1513_ZP | 0 | 0 | 0 | 0.18 | 0.87 | 0.81 |
| c2178/f5p0/1771_ZPZC | 5.92 | 3.78 | 3.49 | 18.27 | 14.49 | 12.93 |
| c20355/f1p1/1848_ZPZC | 0 | 0 | 0.01 | 114.84 | 79.83 | 72.19 |
| c100952/f4p0/1241_ZPZC | 0 | 0 | 0 | 69.85 | 86.56 | 68.27 |
| c40544/f2p3/1238_ZPZC | 0 | 0 | 0 | 22.07 | 21.42 | 20.09 |
| c101154/f3p1/1505_ZPZC | 0 | 0 | 0 | 6.49 | 0.08 | 31.46 |
| c86020/f1p3/826_ZPZC | 0 | 0 | 0 | 0 | 13.2 | 24.98 |
| c42460/f1p0/1354_ZPZC | 0 | 0 | 0.58 | 33.8 | 5.58 | 2.58 |
| c3114/f1p0/2683_YS | 0.09 | 0.95 | 0.51 | 3.29 | 4.84 | 3.35 |
| c1392/f6p0/2414_ZP | 39.22 | 42.4 | 18.17 | 242.55 | 225.28 | 165.23 |
| c4038/f1p2/2630_YS | 11.24 | 13.75 | 7.11 | 62.12 | 55.88 | 32.95 |
| c49401/f1p4/1689_ZPZC | 14.77 | 12.39 | 7.48 | 56.14 | 34.03 | 24.67 |
| c10920/f1p1/2770_ZP | 2.3 | 2.79 | 3.03 | 9.52 | 8.39 | 4.77 |
| c868/f6p0/1699_ZPZC | 5.48 | 5.2 | 4.15 | 35.95 | 20.73 | 10.81 |
| c46788/f1p2/1649_ZPZC | 12.33 | 14.12 | 10.15 | 31.96 | 35.5 | 34.82 |
| c223029/f1p0/1208_YS | 0 | 0 | 0 | 15.38 | 64.94 | 0.1 |
| c270786/f15p1/1176_YS | 1.59 | 2.36 | 3.68 | 50.27 | 9.15 | 23.47 |
| c208236/f3p0/1304_YS | 2 | 0.44 | 2.76 | 21.23 | 14.51 | 13.56 |
| c42049/f1p0/1464_YS | 5.53 | 5.14 | 8.35 | 18.75 | 14.96 | 12.67 |
| c2712/f1p4/1371_ZPZC | 0 | 0 | 0 | 0.86 | 2.44 | 1.04 |
| c32731/f1p4/1164_ZPZC | 0.28 | 0 | 0.23 | 2.82 | 1.51 | 1.95 |
| c14970/f2p0/1368_ZP | 17.41 | 10.74 | 16.25 | 36.04 | 66.06 | 46.45 |
| c10393/f2p1/2024_YS | 0.68 | 2.24 | 2.23 | 14.34 | 10.14 | 9.03 |
| c84658/f1p2/1193_ZPZC | 0 | 0 | 0.28 | 34.72 | 126.32 | 85.82 |
| c7141/f1p1/1084_YS | 0 | 0 | 0 | 3.99 | 19.87 | 9.6 |
| c13151/f4p0/1872_ZP | 0 | 0 | 0 | 3.13 | 6.25 | 3.99 |
| c19656/f2p3/1728_ZPZC | 5.41 | 2.59 | 2.15 | 74.58 | 59.62 | 45.46 |
| c3547/f1p0/1086_ZP | 18.87 | 3.87 | 9.88 | 112.68 | 73.78 | 90.89 |
| c12244/f1p0/1638_ZPZC | 0.24 | 0.14 | 0.31 | 3.12 | 0.6 | 1.42 |
| c4898/f1p1/951_ZPZC | 0 | 0 | 0.19 | 45.15 | 4.83 | 2.89 |
| c174559/f1p0/2150_YS | 0 | 0 | 0 | 0.97 | 0.18 | 0.59 |
| c70349/f1p1/1158_ZPZC | 0 | 0 | 0.29 | 3.67 | 1.68 | 1.72 |
| c12185/f1p1/1200_ZPZC | 0.21 | 0.25 | 1.17 | 13.72 | 11 | 6.8 |
| c22261/f1p1/1664_ZPZC | 0.47 | 0.99 | 14.01 | 90.9 | 72.59 | 44.54 |
| c9442/f1p0/1340_ZP | 0.25 | 0.23 | 1.2 | 7.24 | 4.4 | 3.44 |
| c12930/f1p0/1380_ZP | 1.36 | 0.98 | 0.98 | 5.33 | 2.82 | 5.06 |
| c51966/f1p0/1690_ZPZC | 0.34 | 0.62 | 0.56 | 6.12 | 2.28 | 2.55 |
| c41588/f5p0/1491_ZP | 0 | 0 | 0 | 156.71 | 126.12 | 101.61 |
| c102549/f1p3/937_ZPZC | 0 | 0 | 0.36 | 52.44 | 70.06 | 69.22 |
| c39162/f6p4/1448_ZPZC | 10.5 | 9.38 | 17.46 | 48.47 | 25.19 | 34.13 |
| c26405/f1p0/1686_ZPZT | 11.96 | 10.73 | 15.24 | 33.79 | 37.37 | 23.16 |
| c86745/f1p0/821_YS | 0 | 0 | 0 | 27.16 | 0 | 0 |
| c112201/f1p0/1591_YS | 0 | 0 | 0 | 0 | 5.37 | 0 |
| c28038/f1p5/1383_ZPZC | 0 | 0 | 0 | 17.01 | 0 | 0 |
| c41745/f4p0/1268_ZP | 0 | 0 | 0 | 69.09 | 91.19 | 124.4 |
| c105199/f1p0/656_ZPZC | 0 | 0 | 0 | 91.76 | 167.27 | 298.34 |
| c270553/f1p1/960_YS | 0 | 0 | 0 | 53.94 | 85.18 | 128.05 |
| c34118/f1p1/789_ZPZC | 0 | 0 | 0 | 5.21 | 34.38 | 58.23 |
| c26756/f1p1/1197_YS | 0 | 0 | 0 | 0 | 33.49 | 14.07 |
| c40133/f2p2/1467_ZPZC | 0 | 0 | 0 | 0 | 30.92 | 4.07 |
| c270956/f26p0/1681_YS | 0 | 0 | 0.54 | 98.92 | 103.53 | 56.83 |
| c10292/f1p1/1652_ZP | 0 | 0 | 0 | 0.48 | 2.12 | 0.54 |
| c41417/f1p5/1767_ZPZC | 0.87 | 1.06 | 1.6 | 11.63 | 19.52 | 41.46 |
| c48717/f1p1/1953_ZPZC | 0 | 0 | 0 | 15.47 | 1.67 | 1.03 |
| c52629/f1p3/3108_ZPZC | 0.12 | 0.23 | 0.22 | 1.26 | 3.17 | 3.76 |
| c25635/f1p1/1013_ZP | 2.03 | 2.45 | 3.18 | 13 | 32.76 | 33.54 |
| c15739/f1p0/1631_ZPZC | 3.07 | 1.16 | 3.14 | 10.32 | 10.32 | 7.62 |
| c12117/f1p0/2093_ZPZC | 1.41 | 1.57 | 2.42 | 6.65 | 4 | 4.5 |
| c20820/f2p0/1943_ZP | 2.6 | 4.72 | 2.79 | 11.13 | 9.94 | 5.81 |
| c12685/f2p0/1154_ZPZC | 0 | 0 | 0 | 0 | 56.35 | 19.98 |
| c51533/f1p0/2051_ZPZT | 0.43 | 1.01 | 0.63 | 2.79 | 3.18 | 2.11 |
| c120201/f1p0/1165_YS | 2.35 | 1.45 | 2.73 | 7.07 | 7.81 | 4.73 |
| c3592/f1p0/2969_ZPZC | 1.59 | 1.06 | 1.68 | 4.41 | 4.76 | 3.21 |

**Table S6** The correlation analysis between transcript isoforms and phthalides

|  | ligustilide | senkyunolide H | senkyunolide I | butylidenephthalide | butylphthalide | senyunolide.A |
| --- | --- | --- | --- | --- | --- | --- |
| c73776/f3p0/2391_YS | 0.94 | 0.71 | 0.71 | 0.49 | 0.83 | 0.60 |
| c25107/f2p0/1677_YS | 0.94 | 0.49 | 0.49 | 0.37 | 0.71 | 0.60 |
| c66845/f2p0/2186_YS | 0.65 | -0.13 | -0.13 | 0.39 | 0.13 | 0.13 |
| c86518/f3p3/1525_ZPZC | 0.88 | 0.58 | 0.58 | 0.21 | 0.76 | 0.76 |
| c24427/f1p3/1899_ZPZC | 0.88 | 0.52 | 0.52 | 0.15 | 0.76 | 0.76 |
| c33491/f1p0/2070_ZPZT | 0.94 | 0.54 | 0.54 | 0.43 | 0.71 | 0.60 |
| c1490/f3p2/2316_ZPZC | 0.94 | 0.71 | 0.71 | 0.49 | 0.83 | 0.60 |
| c48858/f1p0/1024_ZP | 0.66 | 0.54 | 0.54 | -0.20 | 0.77 | 0.89 |
| c107121/f1p0/1718_ZPZC | 0.94 | 0.46 | 0.46 | 0.27 | 0.70 | 0.70 |
| c36451/f1p1/1280_ZPZC | 0.88 | 0.58 | 0.58 | 0.21 | 0.76 | 0.76 |
| c13963/f1p1/1531_YS | 0.83 | 0.37 | 0.37 | 0.14 | 0.60 | 0.71 |
| c42564/f1p2/1350_ZPZC | 0.17 | -0.54 | -0.54 | -0.07 | -0.30 | 0.03 |
| c22794/f1p0/1116_ZP | 0.94 | 0.46 | 0.46 | 0.27 | 0.70 | 0.70 |
| c28447/f1p2/1921_ZPZC | 0.85 | 0.14 | 0.14 | 0.44 | 0.37 | 0.37 |
| c15961/f1p0/995_ZP | 0.68 | 0.30 | 0.30 | 0.10 | 0.54 | 0.54 |
| c12700/f1p2/2098_ZPZC | 0.77 | 0.77 | 0.77 | 0.14 | 0.89 | 0.77 |
| c114409/f1p0/1841_YS | 0.77 | 0.26 | 0.26 | -0.03 | 0.54 | 0.77 |
| c40419/f3p2/1688_ZPZC | 0.71 | 0.31 | 0.31 | -0.14 | 0.60 | 0.83 |
| c18236/f1p2/840_ZPZC | 0.54 | 0.60 | 0.60 | -0.37 | 0.77 | 1.00 |
| c27078/f1p2/1676_ZPZC | 0.93 | 0.64 | 0.64 | 0.38 | 0.81 | 0.64 |
| c82794/f1p0/1954_ZPZT | 0.66 | 0.66 | 0.66 | -0.09 | 0.77 | 0.89 |
| c98537/f1p0/1126_ZPZT | 0.13 | 0.65 | 0.65 | -0.39 | 0.65 | 0.65 |
| c10933/f1p0/1354_ZP | 0.77 | 0.77 | 0.77 | 0.14 | 0.89 | 0.77 |
| c41582/f3p0/2370_YS | 0.83 | 0.89 | 0.89 | 0.31 | 0.94 | 0.71 |
| c172739/f1p0/1655_YS | 0.83 | 0.31 | 0.31 | 0.09 | 0.60 | 0.71 |
| c24347/f1p0/1514_ZP | 1.00 | 0.60 | 0.60 | 0.54 | 0.77 | 0.54 |
| c16431/f1p0/1343_YS | 0.77 | 0.49 | 0.49 | 0.09 | 0.66 | 0.77 |
| c26825/f1p0/1628_ZPZT | 0.77 | 0.49 | 0.49 | 0.09 | 0.66 | 0.77 |
| c49867/f1p0/1149_ZPZC | 0.77 | 0.83 | 0.83 | 0.20 | 0.89 | 0.77 |
| c61565/f1p0/1035_YS | 0.71 | 0.37 | 0.37 | -0.09 | 0.60 | 0.83 |
| c3664/f3p0/1817_YS | 0.77 | 0.37 | 0.37 | -0.03 | 0.66 | 0.77 |
| c61565/f1p0/1035_YS | 0.71 | 0.37 | 0.37 | -0.09 | 0.60 | 0.83 |
| c24590/f1p0/1723_ZPZC | 0.70 | 0.82 | 0.82 | -0.09 | 0.94 | 0.94 |
| c49687/f1p0/1551_ZPZC | 0.13 | 0.65 | 0.65 | -0.39 | 0.65 | 0.65 |
| c2460/f2p1/1076_ZPZC | 0.71 | 0.31 | 0.31 | -0.14 | 0.60 | 0.83 |
| c349510/f1p0/975_YS | 0.39 | 0.39 | 0.39 | 0.13 | 0.39 | 0.39 |
| c53819/f1p0/1923_ZPZT | 0.77 | 0.43 | 0.43 | 0.03 | 0.66 | 0.77 |
| c73461/f1p0/1689_ZPZT | 0.88 | 0.58 | 0.58 | 0.21 | 0.76 | 0.76 |
| c113652/f1p0/1125_YS | 0.88 | 0.58 | 0.58 | 0.21 | 0.76 | 0.76 |
| c70277/f1p0/1551_ZPZC | 0.85 | 0.14 | 0.14 | 0.44 | 0.37 | 0.37 |
| c23271/f1p0/1511_ZPZT | 0.83 | 0.37 | 0.37 | 0.14 | 0.60 | 0.71 |
| c9126/f1p0/1016_ZP | 0.78 | 0.27 | 0.27 | 0.37 | 0.44 | 0.44 |
| c16304/f2p0/1341_ZP | 0.71 | 0.37 | 0.37 | -0.09 | 0.60 | 0.83 |
| c17892/f1p1/873_ZPZC | 0.94 | 0.46 | 0.46 | 0.27 | 0.70 | 0.70 |
| c11197/f1p0/1188_ZP | 0.76 | 0.76 | 0.76 | 0.03 | 0.88 | 0.88 |
| c10154/f1p0/1513_ZP | 0.76 | 0.76 | 0.76 | 0.03 | 0.88 | 0.88 |
| c2178/f5p0/1771_ZPZC | 1.00 | 0.60 | 0.60 | 0.54 | 0.77 | 0.54 |
| c20355/f1p1/1848_ZPZC | 0.81 | 0.32 | 0.32 | 0.06 | 0.58 | 0.75 |
| c100952/f4p0/1241_ZPZC | 0.88 | 0.58 | 0.58 | 0.21 | 0.76 | 0.76 |
| c40544/f2p3/1238_ZPZC | 0.94 | 0.46 | 0.46 | 0.27 | 0.70 | 0.70 |
| c101154/f3p1/1505_ZPZC | 0.76 | 0.70 | 0.70 | -0.03 | 0.88 | 0.88 |
| c86020/f1p3/826_ZPZC | 0.37 | 0.85 | 0.85 | -0.27 | 0.85 | 0.85 |
| c42460/f1p0/1354_ZPZC | 0.81 | 0.32 | 0.32 | 0.06 | 0.58 | 0.75 |
| c3114/f1p0/2683_YS | 0.66 | 0.60 | 0.60 | -0.14 | 0.77 | 0.89 |
| c1392/f6p0/2414_ZP | 0.94 | 0.49 | 0.49 | 0.37 | 0.71 | 0.60 |
| c4038/f1p2/2630_YS | 0.94 | 0.49 | 0.49 | 0.37 | 0.71 | 0.60 |
| c49401/f1p4/1689_ZPZC | 1.00 | 0.60 | 0.60 | 0.54 | 0.77 | 0.54 |
| c10920/f1p1/2770_ZP | 0.77 | 0.26 | 0.26 | -0.03 | 0.54 | 0.77 |
| c868/f6p0/1699_ZPZC | 1.00 | 0.60 | 0.60 | 0.54 | 0.77 | 0.54 |
| c46788/f1p2/1649_ZPZC | 0.77 | 0.77 | 0.77 | 0.14 | 0.89 | 0.77 |
| c223029/f1p0/1208_YS | 0.88 | 0.58 | 0.58 | 0.21 | 0.76 | 0.76 |
| c270786/f15p1/1176_YS | 0.71 | 0.31 | 0.31 | -0.14 | 0.60 | 0.83 |
| c208236/f3p0/1304_YS | 0.83 | 0.37 | 0.37 | 0.14 | 0.60 | 0.71 |
| c42049/f1p0/1464_YS | 0.83 | 0.37 | 0.37 | 0.14 | 0.60 | 0.71 |
| c2712/f1p4/1371_ZPZC | 0.76 | 0.76 | 0.76 | 0.03 | 0.88 | 0.88 |
| c32731/f1p4/1164_ZPZC | 0.89 | 0.60 | 0.60 | 0.31 | 0.77 | 0.66 |
| c14970/f2p0/1368_ZP | 0.77 | 0.83 | 0.83 | 0.20 | 0.89 | 0.77 |
| c10393/f2p1/2024_YS | 0.83 | 0.31 | 0.31 | 0.09 | 0.60 | 0.71 |
| c84658/f1p2/1193_ZPZC | 0.64 | 0.61 | 0.61 | -0.17 | 0.75 | 0.93 |
| c7141/f1p1/1084_YS | 0.76 | 0.76 | 0.76 | 0.03 | 0.88 | 0.88 |
| c13151/f4p0/1872_ZP | 0.76 | 0.76 | 0.76 | 0.03 | 0.88 | 0.88 |
| c19656/f2p3/1728_ZPZC | 1.00 | 0.60 | 0.60 | 0.54 | 0.77 | 0.54 |
| c3547/f1p0/1086_ZP | 0.89 | 0.60 | 0.60 | 0.31 | 0.77 | 0.66 |
| c12244/f1p0/1638_ZPZC | 0.77 | 0.43 | 0.43 | 0.03 | 0.66 | 0.77 |
| c4898/f1p1/951_ZPZC | 0.81 | 0.32 | 0.32 | 0.06 | 0.58 | 0.75 |
| c174559/f1p0/2150_YS | 0.88 | 0.52 | 0.52 | 0.15 | 0.76 | 0.76 |
| c70349/f1p1/1158_ZPZC | 0.75 | 0.38 | 0.38 | -0.06 | 0.64 | 0.81 |
| c12185/f1p1/1200_ZPZC | 0.77 | 0.26 | 0.26 | -0.03 | 0.54 | 0.77 |
| c22261/f1p1/1664_ZPZC | 0.77 | 0.26 | 0.26 | -0.03 | 0.54 | 0.77 |
| c9442/f1p0/1340_ZP | 0.83 | 0.37 | 0.37 | 0.14 | 0.60 | 0.71 |
| c12930/f1p0/1380_ZP | 0.93 | 0.64 | 0.64 | 0.38 | 0.81 | 0.64 |
| c51966/f1p0/1690_ZPZC | 0.77 | 0.37 | 0.37 | -0.03 | 0.66 | 0.77 |
| c41588/f5p0/1491_ZP | 0.94 | 0.46 | 0.46 | 0.27 | 0.70 | 0.70 |
| c102549/f1p3/937_ZPZC | 0.64 | 0.61 | 0.61 | -0.17 | 0.75 | 0.93 |
| c39162/f6p4/1448_ZPZC | 0.77 | 0.43 | 0.43 | 0.03 | 0.66 | 0.77 |
| c26405/f1p0/1686_ZPZT | 0.77 | 0.49 | 0.49 | 0.09 | 0.66 | 0.77 |
| c86745/f1p0/821_YS | 0.65 | -0.13 | -0.13 | 0.39 | 0.13 | 0.13 |
| c112201/f1p0/1591_YS | 0.39 | 0.39 | 0.39 | 0.13 | 0.39 | 0.39 |
| c28038/f1p5/1383_ZPZC | 0.65 | -0.13 | -0.13 | 0.39 | 0.13 | 0.13 |
| c41745/f4p0/1268_ZP | 0.70 | 0.82 | 0.82 | -0.09 | 0.94 | 0.94 |
| c105199/f1p0/656_ZPZC | 0.70 | 0.82 | 0.82 | -0.09 | 0.94 | 0.94 |
| c270553/f1p1/960_YS | 0.70 | 0.82 | 0.82 | -0.09 | 0.94 | 0.94 |
| c34118/f1p1/789_ZPZC | 0.70 | 0.82 | 0.82 | -0.09 | 0.94 | 0.94 |
| c26756/f1p1/1197_YS | 0.44 | 0.78 | 0.78 | -0.14 | 0.78 | 0.78 |
| c40133/f2p2/1467_ZPZC | 0.44 | 0.78 | 0.78 | -0.14 | 0.78 | 0.78 |
| c270956/f26p0/1681_YS | 0.75 | 0.43 | 0.43 | 0.00 | 0.64 | 0.81 |
| c10292/f1p1/1652_ZP | 0.76 | 0.76 | 0.76 | 0.03 | 0.88 | 0.88 |
| c41417/f1p5/1767_ZPZC | 0.54 | 0.60 | 0.60 | -0.37 | 0.77 | 1.00 |
| c48717/f1p1/1953_ZPZC | 0.94 | 0.46 | 0.46 | 0.27 | 0.70 | 0.70 |
| c52629/f1p3/3108_ZPZC | 0.60 | 0.66 | 0.66 | -0.26 | 0.83 | 0.94 |
| c25635/f1p1/1013_ZP | 0.54 | 0.60 | 0.60 | -0.37 | 0.77 | 1.00 |
| c15739/f1p0/1631_ZPZC | 0.81 | 0.43 | 0.43 | 0.12 | 0.64 | 0.75 |
| c12117/f1p0/2093_ZPZC | 0.71 | 0.31 | 0.31 | -0.14 | 0.60 | 0.83 |
| c20820/f2p0/1943_ZP | 0.83 | 0.31 | 0.31 | 0.09 | 0.60 | 0.71 |
| c12685/f2p0/1154_ZPZC | 0.44 | 0.78 | 0.78 | -0.14 | 0.78 | 0.78 |
| c51533/f1p0/2051_ZPZT | 0.77 | 0.43 | 0.43 | 0.03 | 0.66 | 0.77 |
| c120201/f1p0/1165_YS | 0.77 | 0.49 | 0.49 | 0.09 | 0.66 | 0.77 |
| c3592/f1p0/2969_ZPZC | 0.77 | 0.49 | 0.49 | 0.09 | 0.66 | 0.77 |

**Table S7** UPLC-ESI-MS/MS method validation parameters for the tested components of MEJA treatment samples (n = 6)

| Analytes | Regression  Equation | R^2^ | Linear Range (µg/mL) | LOD (ng/mL) | LOQ (ng/mL) | Precision  (RSD, %) | Stability  (RSD, %) | Reproducibility  (RSD, %) | Recovery | |
| --- | --- | --- | --- | --- | --- | --- | --- | --- | --- | --- |
|  |  |  |  |  |  |  |  |  | Mean, % | RSD, % |
| ligustilide | y=6.788×10^6^x+3.719×10^6^ | 0.994 | 0.0227-23.20 | 0.4201 | 1.401 | 1.22 | 1.44 | 1.68 | 99.17 | 1.75 |
| senkyunolide H | y=2.870×10^7^x+1.449×10^5^ | 0.999 | 0.0069-1.766 | 0.6146 | 2.049 | 1.79 | 2.24 | 2.42 | 98.93 | 1.59 |
| senkyunolide I | y=2.526×10^7^x+3.034×10^6^ | 0.991 | 0.0143-7.313 | 0.6878 | 2.293 | 1.06 | 1.78 | 1.87 | 100.2 | 1.47 |
| butylidenephthalide | y=5.150×10^7^x+9.783×10^6^ | 0.991 | 0.0084-8.656 | 0.0840 | 0.2801 | 1.28 | 2.25 | 2.52 | 101.0 | 1.70 |
| butylphthalide | y=8.186×10^7^x+4.221×10^6^ | 0.993 | 0.0083-2.129 | 0.1048 | 0.3493 | 1.48 | 1.32 | 1.82 | 100.7 | 1.80 |
| senyunolide A | y=1.122×10^8^x+1.462×10^7^ | 0.990 | 0.0598-1.914 | 0.0279 | 0.0930 | 1.53 | 2.48 | 2.34 | 98.66 | 1.82 |

**Table S8** Summary information of 7 candidate isoforms

| NO | Isoform_id | length | NR_protein_accession | NR_defination | Pathway_ID |
| --- | --- | --- | --- | --- | --- |
| 17 | c114409/f1p0/1841_YS | EC 2.5.1.54 | XP_017246864.1 | PREDICTED: phospho-2-dehydro-3-deoxyheptonate aldolase 2, chloroplastic [Daucus carota subsp. sativus] | ko00400 |
| 21 | c82794/f1p0/1954_ZPZT | EC 4.2.1.10 | XP_017220070.1 | PREDICTED: bifunctional 3-dehydroquinate dehydratase/shikimate dehydrogenase, chloroplastic-like isoform X2 [Daucus carota subsp. sativus] | ko00400 |
| 23 | c98537/f1p0/1126_ZPZT | EC 1.4.3.21 | XP_017235835.1 | PREDICTED: primary amine oxidase-like [Daucus carota subsp. sativus] | ko00400 |
| 24 | c10933/f1p0/1354_ZP | EC 1.4.3.21 | XP_017253318.1 | PREDICTED: primary amine oxidase-like [Daucus carota subsp. sativus] | ko00960 |
| 36 | c49687/f1p0/1149_ZPZC | EC 1.10.3.1 | XP_017232346.1 | PREDICTED: polyphenol oxidase, chloroplastic-like [Daucus carota subsp. sativus] | ko00350 |
| 38 | c26825/f1p0/1628_ZPZT | EC 4.1.1.25 | AAA33861.1 | tyrosine decarboxylase, partial [Petroselinum crispum] | ko00350 |
| 43 | c113652/f1p0/1125_YS | EC 2.3.1.133 | XP_017252778.1 | PREDICTED: shikimate O-hydroxycinnamoyltransferase [Daucus carota subsp. sativus] | ko00940 |


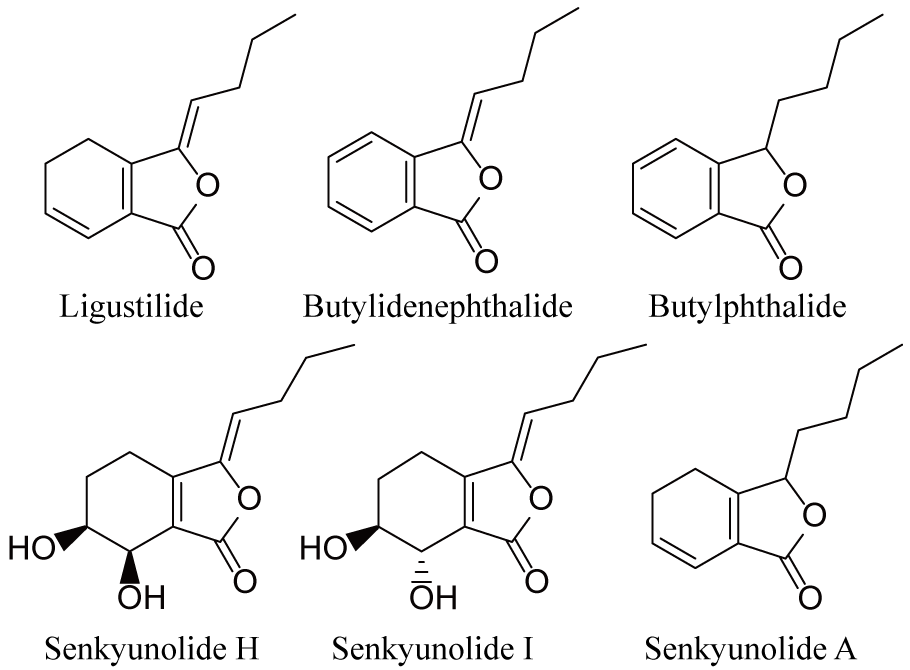


**Figure S1** Chemical structures of six tested phathalides in *A. sinensis*


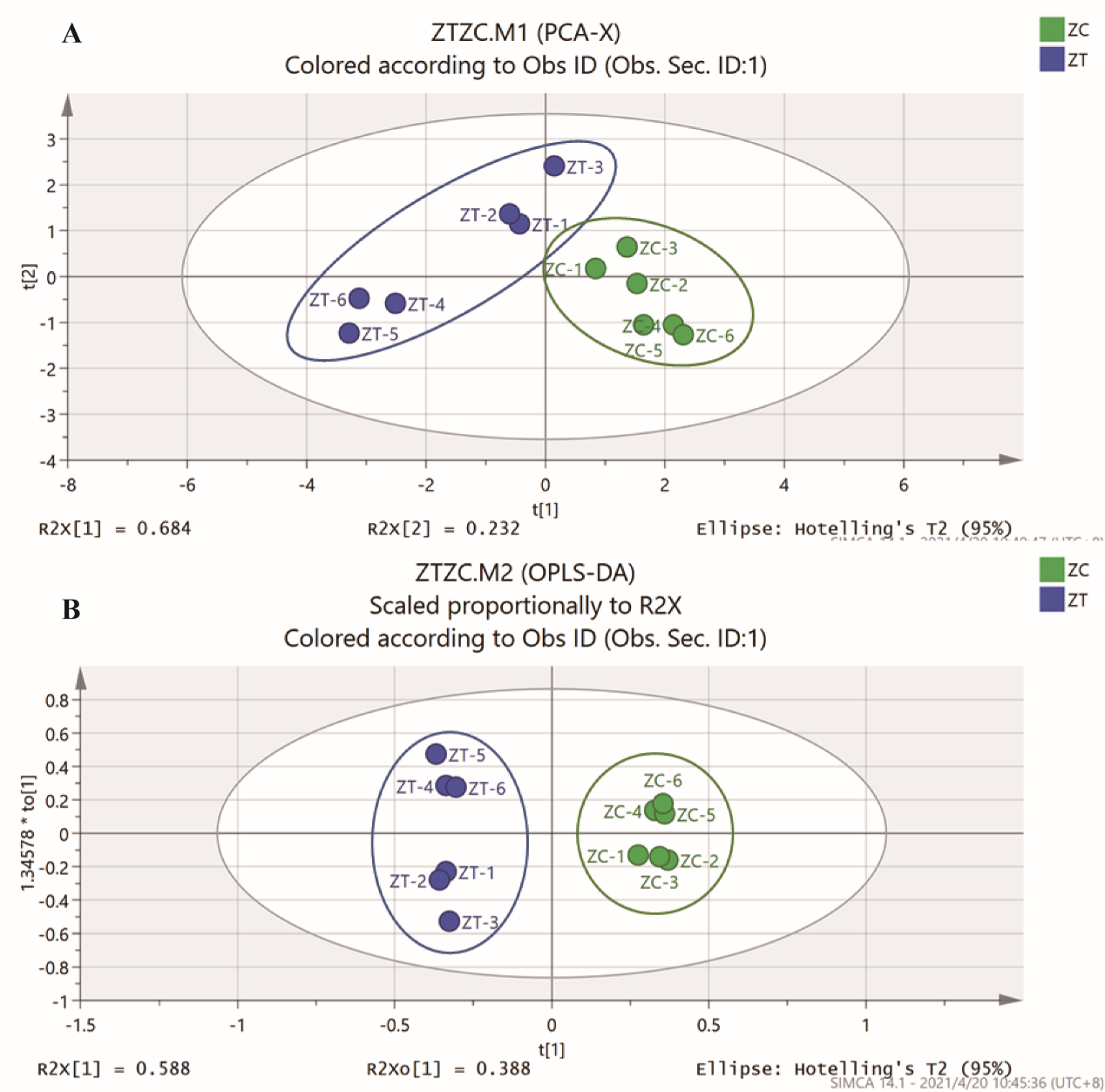


**Figure S2** Multivariate statistical analysis of methanol extracts of ZT and ZC samples (A: PCA scores plot; B: OPLS-DA scores plot.)


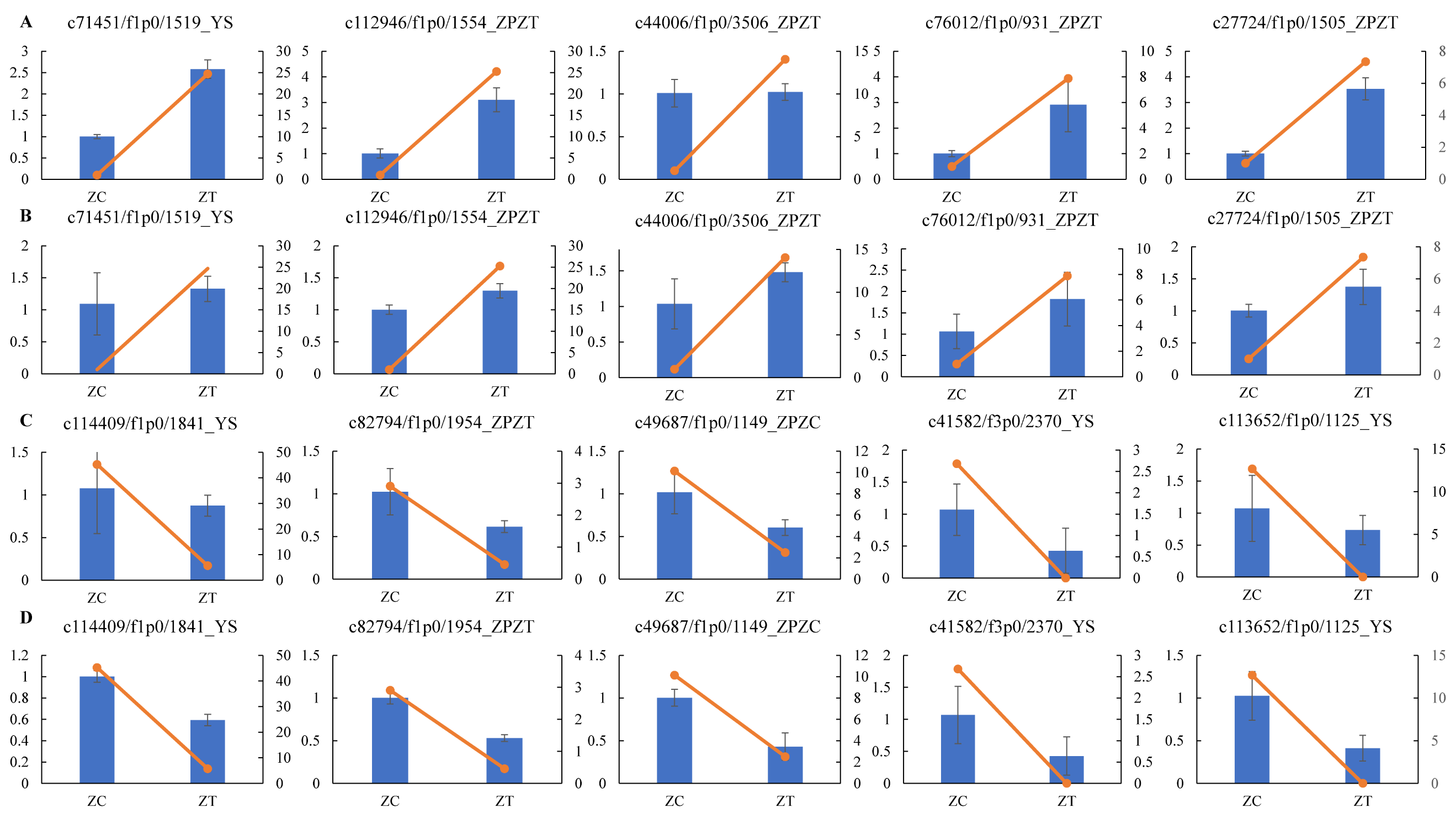


**Figure S3** The verification of the expression of 10 isoforms by qRT-PCR. (Blue histogram: qRT-PCR; Orange line: RNA-seq). The left Y-axis indicates the expression of 10 isoforms by qRT-PCR, and the right Y-axis indicates the expression of 10 isoforms by RNA-seq. A and C were normalized to 18s-RNA; B and D were normalized to actin


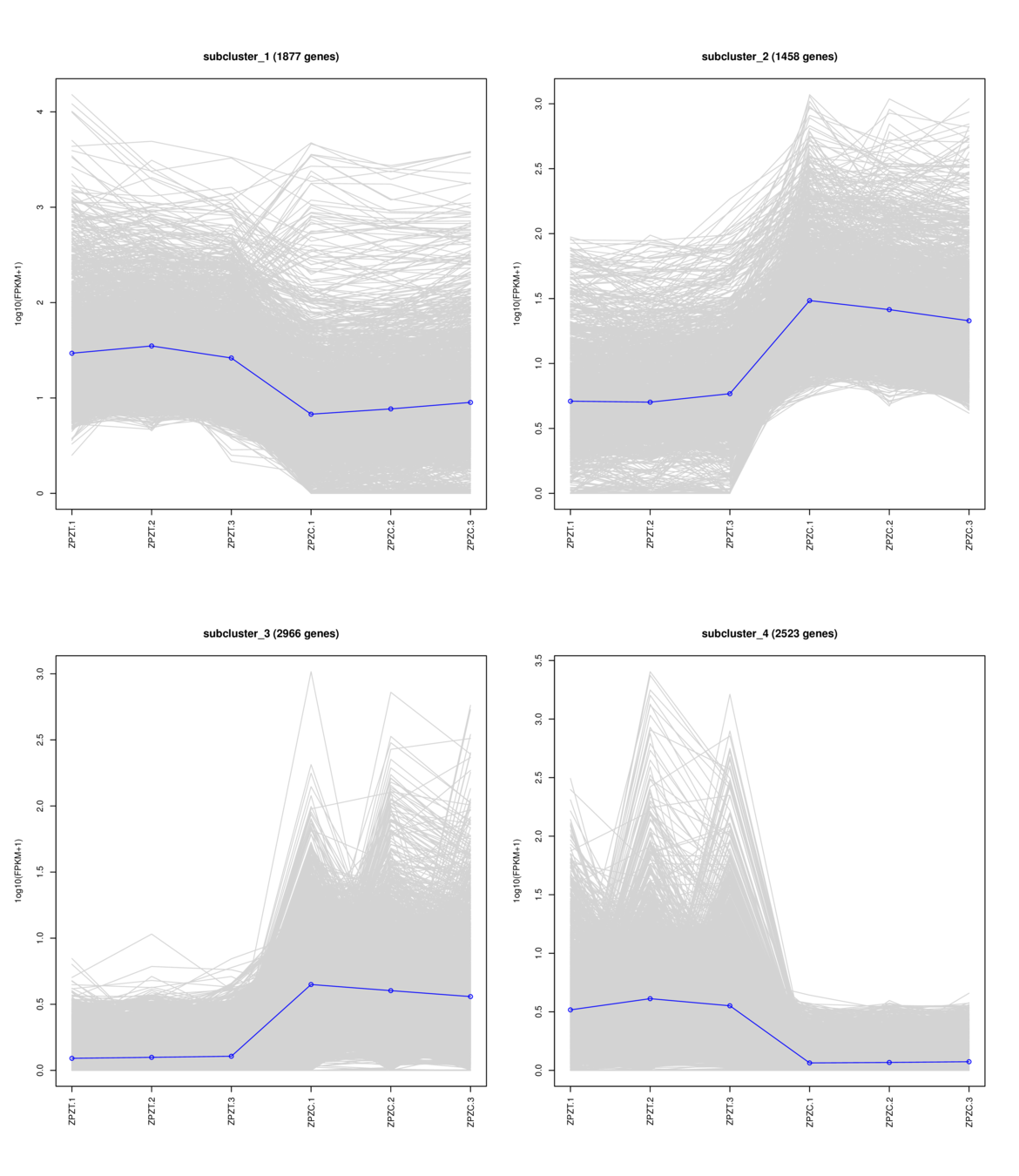


**Figure S4** Relative expression of differentially expressed isoforms in different subcluster of ZC and ZT samples. (The gray line indicates the relative expression of transcripts in this cluster at different samples, and the blue line indicates the average relative expression of all transcripts in this cluster at different samples. The Y-axis indicates log^10(FPKM+1)^; the X-axis indicates different samples.)


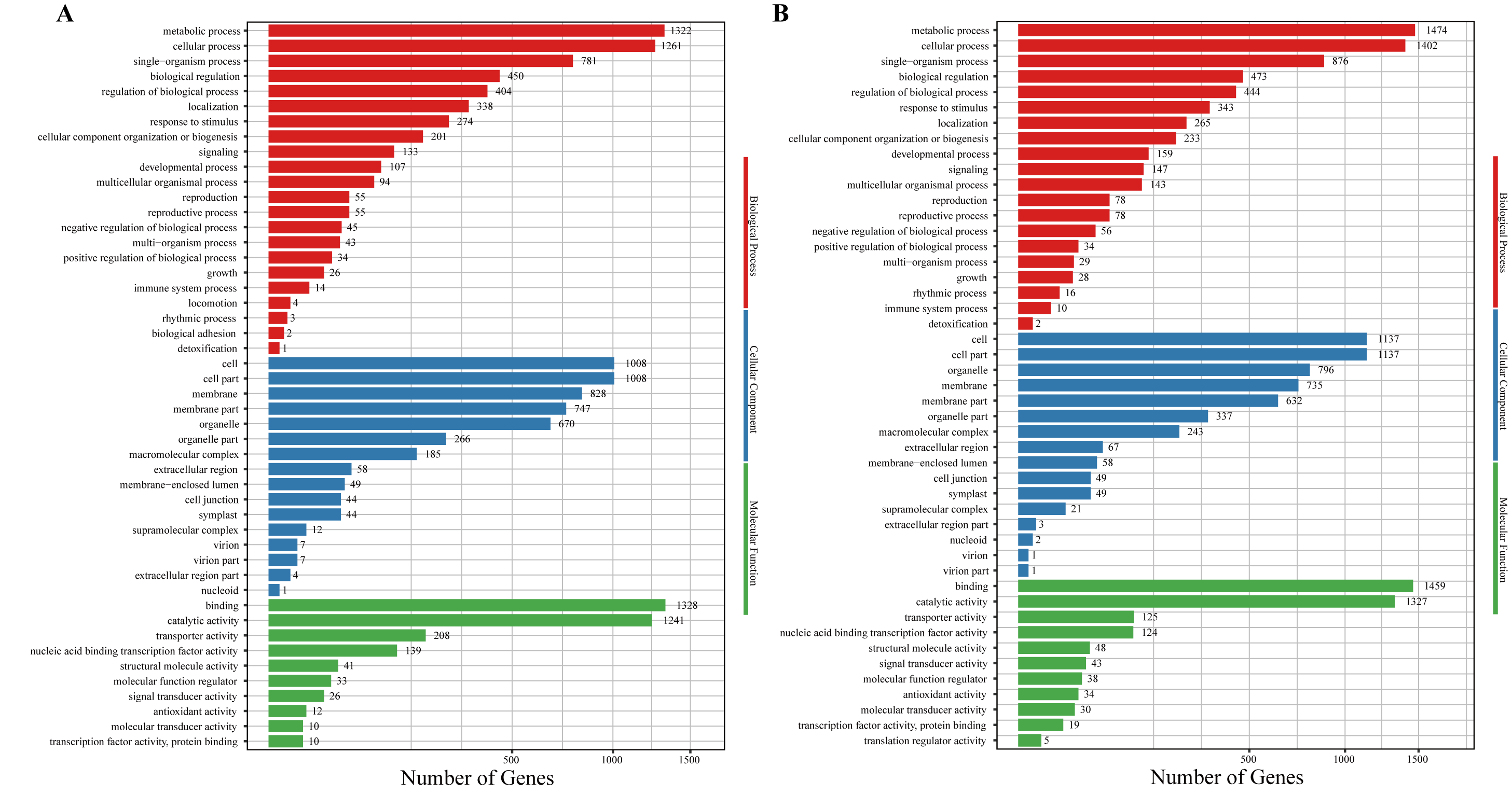


**Figure S5** Gene ontology (GO) enrichment analysis of up- and downregulated isoforms identified in ZT vs ZC (A: downregulated; B: upregulated). The Y-axis indicates the top enriched GO terms; the X-axis indicates the number of genes annotated to corresponding term to the total number of genes annotated.


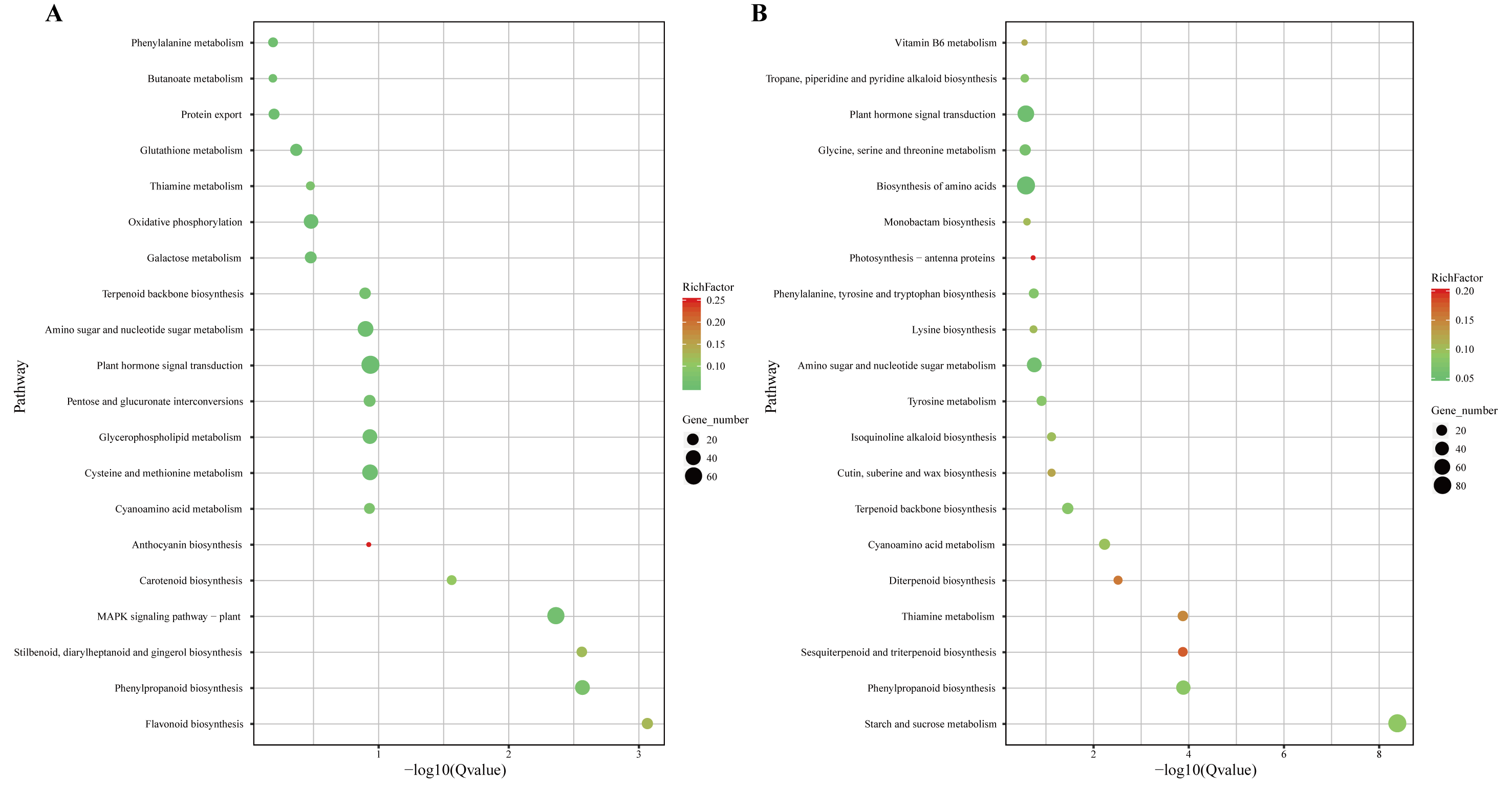


**Figure S6** KEGG pathway enrichment analysis of the annotated isoforms in ZT vs ZC (A: downregulated; B: upregulated). The Y-axis indicates the enriched KEGG pathway, and the X-axis indicates the rich factor. The dot size indicates the number of isoforms in the pathway, and the dot color indicates the q-value


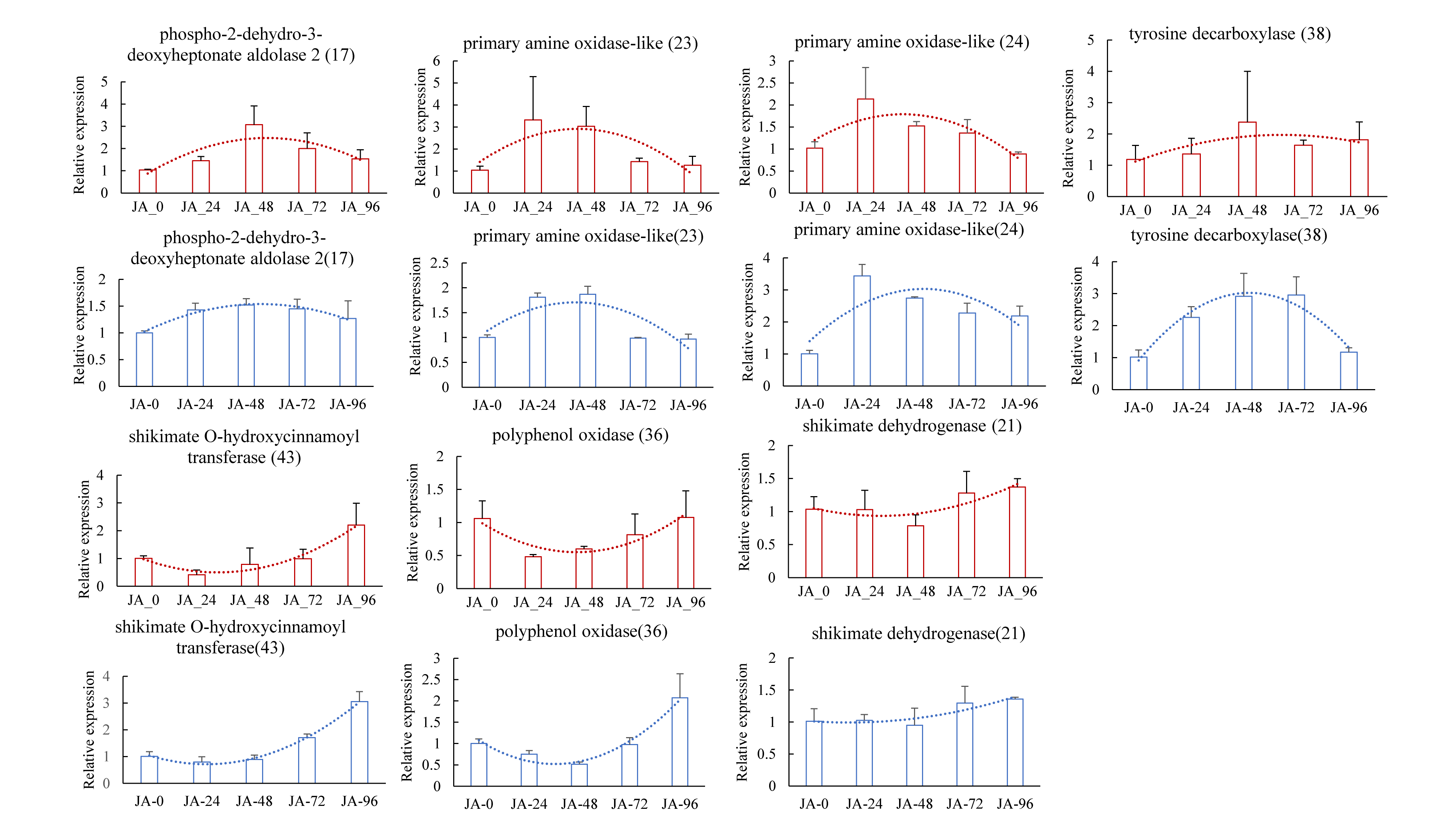


**Figure S7** The relative expression of the isoforms across samples by MeJA treatment (ANOVA: P<0.05; JA-0、24、48、72、96 means tissue culture seedlings of *A. sinensis* were collected at the indicated time points (0h, 24h, 48h, 72h, 96h) after MeJA treatment; Red means the relative expression were normalized to 18s-RNA, blue means the relative expression were normalized to actin.)


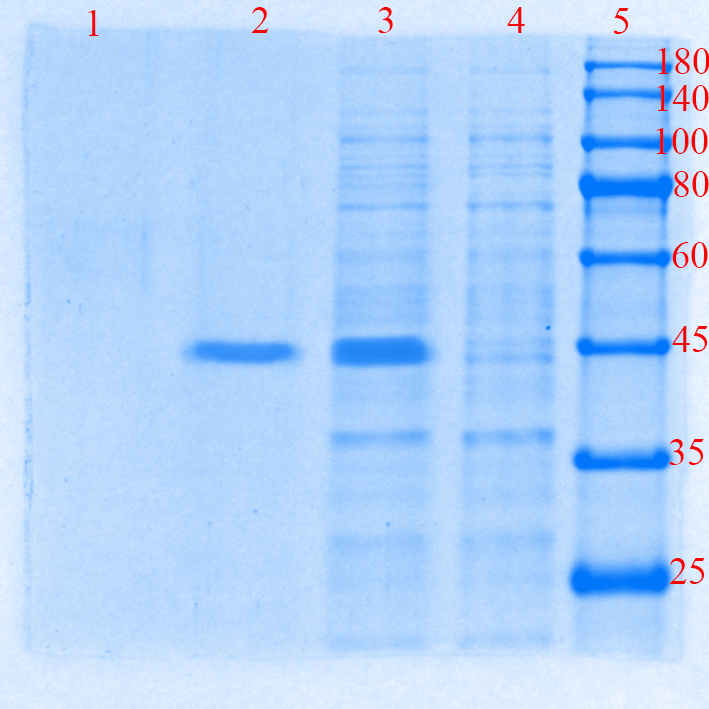


**Figure S8** Expression and purification of shikimate *O*-hydroxycinnamoyl transferase. Proteins were analyzed using SDS PAGE, stained with Coomassie blue (1: Prcipitation; 2: Sample after purification; 3: Post-induction; 4: Pre-induction; 5: marker.)


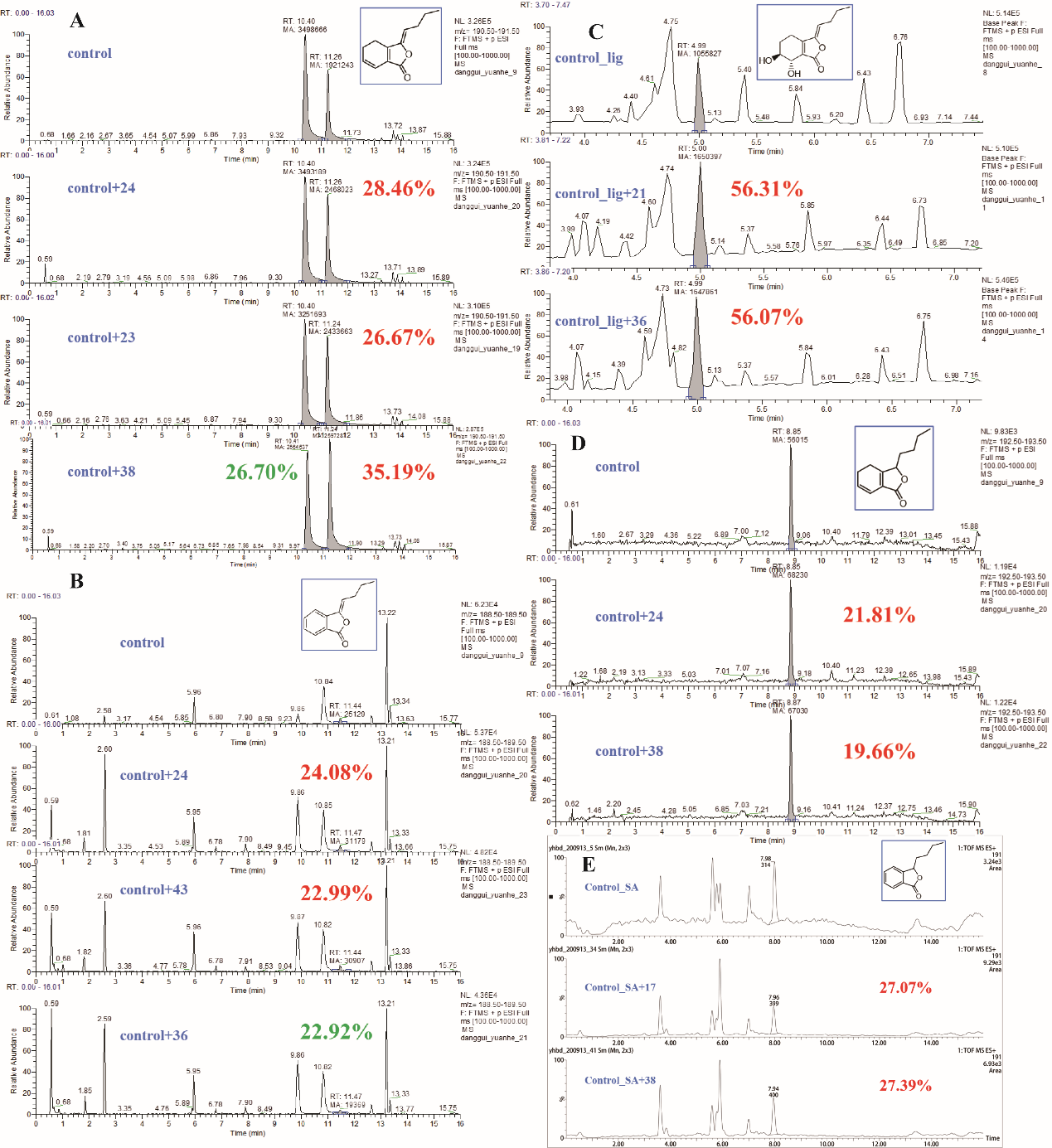


Figure S9 UHPLC-MS/MS analysis of *E. coil* samples incubated with extractions of *A. sinensis* as substrate in vivo. (A: ligustilide; B: butylidenephthalide; C: senkyunolide I; D: senkyunolide A; E: butylphthalide.)
